# Tissue-specific usage of transposable element-derived promoters in mouse development

**DOI:** 10.1186/s13059-020-02164-3

**Published:** 2020-09-28

**Authors:** Benpeng Miao, Shuhua Fu, Cheng Lyu, Paul Gontarz, Ting Wang, Bo Zhang

**Affiliations:** 1 Department of Developmental Biology, Center of Regenerative Medicine, Washington University School of Medicine, St. Louis, MO 63108 USA; 2 Department of Genetics, Edison Family Center for Genomic Sciences and Systems Biology, McDonnell Genome Institute, Washington University School of Medicine, St. Louis, MO 63108 USA

**Keywords:** Accessible transposable element, Mouse, Embryo development, Tissues

## Abstract

**Background:**

Transposable elements (TEs) are a significant component of eukaryotic genomes and play essential roles in genome evolution. Mounting evidence indicates that TEs are highly transcribed in early embryo development and contribute to distinct biological functions and tissue morphology.

**Results:**

We examine the epigenetic dynamics of mouse TEs during the development of five tissues: intestine, liver, lung, stomach, and kidney. We found that TEs are associated with over 20% of open chromatin regions during development. Close to half of these accessible TEs are only activated in a single tissue and a specific developmental stage. Most accessible TEs are rodent-specific. Across these five tissues, 453 accessible TEs are found to create the transcription start sites of downstream genes in mouse, including 117 protein-coding genes and 144 lincRNA genes, 93.7% of which are mouse-specific. Species-specific TE-derived transcription start sites are found to drive the expression of tissue-specific genes and change their tissue-specific expression patterns during evolution.

**Conclusion:**

Our results suggest that TE insertions increase the regulatory potential of the genome, and some TEs have been domesticated to become a crucial component of gene and regulate tissue-specific expression during mouse tissue development.

## Background

In the mammalian genome, only about 2% of DNA can be translated into protein products; the remaining ~ 98% of non-coding genome is considered to be “genomic dark matter” with unknown function [1–3]. Within these non-coding sequences, approximately 37% of the mouse genome and 45% of the human genome are derived from different kinds of transposable elements (TEs), including LINE, SINE, ERV, and DNA transposons [1–6]. TEs are highly repetitive DNA units and can reproduce themselves in the host genome. TEs generally belong to two main categories: DNA transposons, which mobilize themselves in the genome through a “cut and paste” mechanism; and retrotransposons, which replicate themselves by a “copy and paste” mechanism and can reach to hundreds of thousand copies in the genome [6, 7].

As parasites in the mammalian genome, TEs can regulate their activities by hijacking the regulatory mechanism of the host genome. Additionally, because of the highly abundant transcriptional factor binding sites in TE sequences, TEs also present a substantial regulatory potential to the host genome [8–11]. In order to resist the damaging effect of TEs, the host genome has evolved multiple mechanisms, in particular epigenetic repression, including DNA methylation and repressive histone methylation, to suppress the activity of TEs in the cell [12]. In the mammalian genome, most TEs are believed to be epigenetically silenced in somatic cells. However, mounting evidence suggests that some TEs escape from epigenetic silencing and are actively involved in a diverse array of biological processes (e.g., embryogenesis and carcinogenesis) and can become an essential component (e.g., promoter, enhancer, or insulator) of gene regulatory networks in the host genome [5, 7–10, 13–17]. Specifically, TEs have been found to initiate some genes’ transcription, especially for genes involved in immunity or response to external stimuli [18–23]. Recent evidence also suggests that TEs are significant contributors to the origin of vertebrate long non-coding RNAs [7, 24]. Meanwhile, TEs were also found to play roles as promoters in early development [25–29] and some terminally differentiated tissues [30, 31].

Activation of silent TEs is tightly associated with epigenetic modification [14, 32–35]. In our previous study [14], we reported the tissue-specific pattern of DNA methylation of 928 TE subfamilies across different human tissues. When TEs were unmethylated in a specific tissue, active enhancer histone modification marks, such as H3K4me1 and H3K27ac, were also enriched around these unmethylated TEs. We further found that these tissue-specific activated TEs can be associated with tissue-specific transcription factors (TFs), suggesting that the TFs might directly control the activation of TEs in a tissue-specific manner. We previously utilized the tissue-specific epigenetic landscape to detect the activation of TEs in human cancer cells [13, 14, 17, 35, 36]. In particular, we reported highly active cryptic transcription of LTR12 subfamilies when the lung cancer cell line NCI-H1299 underwent treatment of a DNMT inhibitor and an HDAC inhibitor [35]. Other studies also indicated the active roles of TEs in neurodegeneration [37] and systemic lupus erythematosus [38]. Altogether, such evidence suggests a critical need to better understand the function of TEs in different biological processes.

In this study, we analyzed the dynamics of chromatin accessibility in five mouse tissues [39, 40], including intestine, liver, lung, kidney, and stomach, to investigate the involvement and contribution of TEs to gene regulatory networks in mouse embryo development. We identified 73,453 accessible TEs in these five tissues, and ~ 53% of these TEs exhibited dynamic accessibility during tissue development. The activation of TEs was strongly associated with the developmental stage-specific transcription factors. The accessibility of TEs displayed a highly tissue-specific pattern, and genes around accessible TEs were associated with tissue-specific functions. During the development of five mouse tissues, 453 TEs were found to create the new 5′ end TSS of genes, including 117 protein-coding genes and 144 lincRNA genes. 13.8% of these genes with TE-derived TSSs showed a tissue-specific expression pattern. Thus, mouse-specific TEs have been domesticated and provided functional promoters for downstream protein-coding genes and have created a novel tissue-specific expression pattern for these genes. Taken together, our study provides a comprehensive investigation into the contribution of TEs to the regulatory landscape of mouse embryonic tissue development.

## Results

### The tissue-specific pattern of accessible TEs in mouse tissue development

**Fig. 1:**
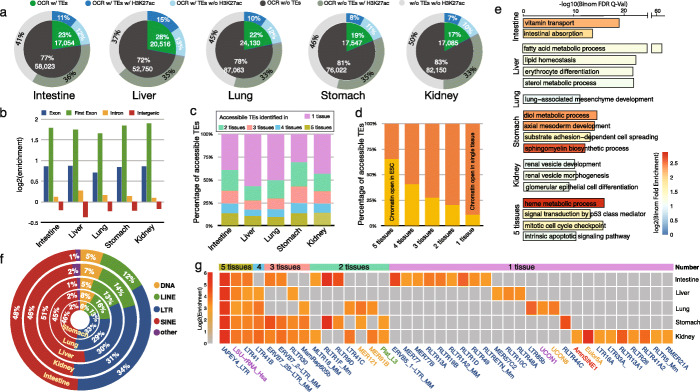
Accessible transposable elements (TEs) in five mouse tissues. **a** Around 17~28% of ATAC-seq peaks identified in five mouse tissues associated with TEs, near half of these accessible TEs recruited H3K27ac signals. **b** Genomic distribution enrichment of accessible TEs of five mouse tissues in the mouse genome. Accessible TEs highly enriched in the exons of the mouse genome, especially the first exon. **c** Distribution of accessible TEs among five mouse tissues. Near half of accessible TEs were identified in only one tissue type. **d** Chromatin status of identified accessible TEs in mouse ES cells. In total, 65% of accessible TEs constitutively in five tissues were accessible in mouse ESC, only 10% of tissue-specific TEs were open in mESC. **e** Gene ontology (biological process) enrichment analysis of tissue-specific accessible TEs in five mouse tissues. GO terms related to tissue-specific function were selected to visualize. **f** TE class distribution of accessible TEs in five mouse tissues. **g** TE subfamily enrichment of accessible TEs in five mouse tissues

To understand the functionality of TEs during mouse embryonic development, we obtained the epigenomic data of five tissues, including intestine, liver, lung, kidney, and stomach, at two developmental stages: embryonic day 14.5 (E14.5) and postnatal day 0 (P0) from the ENCODE [41, 42] data portal (Additional file 1). By analyzing ATAC-seq data from these five mouse tissues, we identified 452,298 open chromatin regions (OCR) in total and examined the accessibility of TEs during tissue development and found ~ 21% of these open chromatin regions were associated with TEs (Additional file 2). Although spatial correlation analysis calculated by GenometriCorr [7, 43] indicated the significant lack of overlap between OCRs and TEs compared with random expectation (Additional file 3), consistent with the general notion that TEs are epigenetically silenced, the sheer number of accessible TEs underscoresd their potential sizable contribution to gene regulation [34, 44, 45]. In each tissue, TEs were associated with 17% (kidney) to 28% (liver) of OCRs, and about half of these accessible TEs directly overlapped with the active histone modification H3K27ac (Fig. 1a; Additional file 4: Table S1). Most accessible TEs (> 90%) were located in intragenic or intergenic regions (Additional file 4: Table S2; Additional file 5: Fig. S1), suggesting that these accessible TEs could be potential enhancers playing a role in tissue development. Interestingly, we noticed that ~ 6% of accessible TEs overlapped with exons and were especially enriched in the first exons comparing with the random background (Fig. 1b). Such results suggested the potential role of TEs as promoters in mouse tissue development as previously reported [7, 19–24, 46–51]. Approximately 10% of accessible TEs were commonly accessible in all five tissues (Fig. 1c), and they were enriched for CTCF, Usf1, and Klf4 binding motifs (Additional file 4: Table S3 and S4). Close to half of accessible TEs were only accessible in a single tissue type (Fig. 1c; Additional file 4: Table S3). These results confirmed previous reports suggesting that the TE activation displayed a strong tissue-specific pattern [14, 24, 28, 52–54].

Next, we asked whether these accessible TEs in developing tissues were also accessible in the mouse embryonic stem cells (mESCs). Interestingly, the majority of the constitutively accessible TEs (65%) already had open chromatin accessibility in mESCs, whereas only 10% of the tissue-specific accessible TEs had open chromatin accessibility in mESCs (Fig. 1d; Additional file 4: Table S5). Thus, the small set of TEs with early and constitutive open chromatin signatures might play a more general regulatory role in development, whereas TEs exhibiting late and tissue-specific open chromatin might be related to tissue-specific gene regulation. Consistent with this hypothesis, our GO enrichment analysis revealed distinct functional enrichment patterns among genes associated with different classes of accessible TEs (Fig. 1e; Additional file 6). The constitutively accessible TEs were more associated with essential biological processes and functions, such as ribosome biogenesis and mitotic cell cycle checkpoint, whereas tissue-specific accessible TEs were more associated with functions directly relevant to the specific tissue types (Fig. 1e; Additional file 6). Close to half of the accessible TEs belonged to the SINE class, and ~ 30% were derived from LTR elements. Of the remaining, 15% were LINEs, and less than 8% of accessible TEs belonged to DNA class (Fig. 1f; Additional file 4: Table S6). However, subfamily enrichment analysis of accessible TEs indicated that most of the top enriched TE subfamilies were LTRs (Fig. 1g; Additional file 4: Table S7). Three TE subfamilies (IAPEY4_LTR, LSU-rRNA_Hsa, and LTR41) were highly enriched in constitutively accessible TEs (Fig. 1g; Additional file 4: Table S7). LTR41b, an LTR subfamily exhibiting strong regulatory ability in human liver cancer cell line HepG2 [55], was highly accessible in four mouse tissues but not the intestine.

### TEs can initiate the transcription of genes during tissue development

**Fig. 2:**
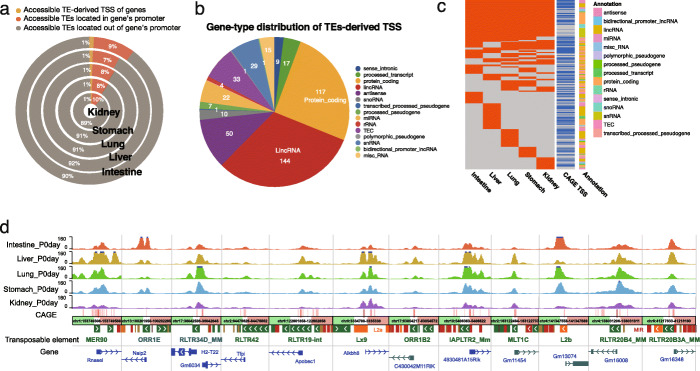
Genes with accessible transposable elements (TEs)-derived TSS in five mouse tissues. **a** Distribution of accessible TEs in the gene’s promoter and TEs-derived TSS in five tissues. Near 10% of accessible TEs located in promoter regions, and 1% accessible TEs contained gene TSS. **b** Gene-type distribution of 453 accessible TE-derived TSSs identified in five mouse tissues, including 117 protein-coding genes and 144 lincRNA. **c** Tissue-specific distribution of 453 accessible TE-derived TSSs. A total of 263 of these accessible TEs can be validated by CAGE-seq. The annotation colors represented different gene types. Near half of TE-derived TSSs were only identified in one tissue. **d** Epigenome browser view of ATAC-seq signals around the accessible TE-derived TSS of protein-coding genes (left six) and lincRNA (right six)

Within the 6% of accessible TEs that overlapped exons, 60% of them overlapped with the first exon of annotated genes (intestine 59.9%; liver 57.5%; lung 60.6%; stomach 62.7%; kidney 64.2%). This result motivated us to examine the relationship between these TEs and the promoter or the transcription start site (TSS) of the overlapping gene. We found ~ 10% of accessible TEs were located in the promoter regions (0.5 kb upstream of the TSS) of genes (Fig. 2a; Additional file 7: Table S1). In particular, we found that about 1% of accessible TEs directly overlapped with the transcription start site of genes, suggesting that TEs might have been domesticated as an integral component of the downstream gene for RNA Polymerase II recruitment and transcription initiation (Fig. 2a). Across the five developing mouse tissues, 453 TEs were found to derive the TSS of genes, including 117 protein-coding genes, 144 lincRNA genes, and 50 antisense genes (Fig. 2b; Additional file 7: Table S2). In total, 58% of these accessible TEs can be independently validated by the FANTOM5 CAGE-TSS signal. A total of 46% of the TEs that can derive the TSS of a gene was only accessible in one tissue, only slightly smaller than the overall tissue specificity of accessible TEs (Fig. 2c; Additional file 7: Table S2). In contrast, ~ 20% of TEs-derived TSSs were constitutively accessible, much higher than the 10% overall constitutively accessible TE percentage (Fig. 2c, d; Additional file 7: Table S2 and S3; https://bit.ly/3hZryCj).

### Dynamically accessible TEs were associated with developmental stage-specific transcription factors

**Fig. 3:**
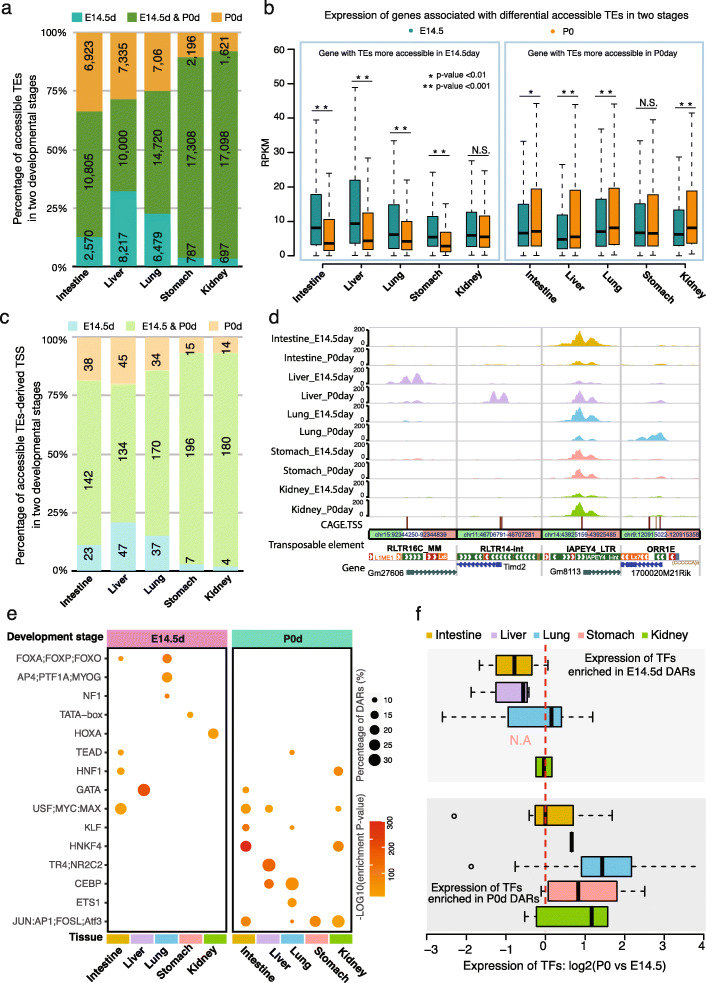
Dynamic accessibility changes of TEs during mouse embryonic development. **a** Percentage of dynamically changed accessible TEs at two development stages in five mouse tissues. E14.5d: the accessible TEs with higher accessibility in the E14.5 development stage; P0d: accessible TEs with higher accessibility in P0day; E14.5&P0d mean accessible TEs did not significantly change their accessibility between the two stages. **b** Expression pattern of genes around accessible TEs at two development stages in five mouse tissues (Wilcoxon signed-rank test). **c** Percentage of dynamically changed accessible TE-derived TSS at two development stages in five mouse tissues. **d** Epigenome browser view of ATAC-seq signals around the dynamically changed accessible TE-derived TSS of protein-coding genes (left three) and lincRNA (right one). **e** Tissue- and developmental stage-specific enrichment of predicted transcription factors binding motifs in dynamically changed accessible TEs at E14.5 (left) and P0 (right) in five mouse tissues. The color of the dot indicated the enrichment *p*-value of motif; the size represented the percentage of DARs that contained the TF motif. **f** Developmental stage-specific expression pattern of TFs that predicted to bind the dynamically changed accessible TEs at E14.5 (top) and P0 (bottom) in five mouse tissues. The dashed line indicated no expression difference of TFs between two stages. N.A: no TF motif was enriched in stomach P0d DARs

Since we incorporated two developmental stages (embryo day 14.5 and postnatal day 0) of five tissues in our analysis, we were able to explore the dynamic accessibility changes of TEs during mouse development. In total, we found that the accessibilities of 38,560 TEs were developmental stage-dependent. In the intestine, liver, and lung, about 50% of accessible TEs showed changes of accessibility between the two developmental stages (Fig. 3a; Additional file 8: Table S1). In contrast, only about 10% of accessible TEs showed changes in the stomach and kidney between E14.5 and P0, suggesting that stage-specific TE accessibilities were also tissue-specific (Fig. 3a). Genes associated with developmental stage-specific accessible TEs generally also displayed developmental stage-specific expression pattern (Fig. 3b). Since ~ 95% of developmental stage-specific accessible TEs were distal to gene’s promoter, our results supported previous findings that TEs might play roles as a distal enhancer in gene regulatory networks (Additional file 8: Table S2) [9, 34, 48, 56]. Furthermore, 214 TE-derived TSSs of genes exhibited accessibility changes (Fig. 3c; Additional file 8: Table S1). In the liver, 47 TE-derived TSSs were accessible only at E14.5, and 45 TE-derived TSSs were accessible at P0. In contrast, the numbers for the kidney were 4 and 14 for E14.5 and P0, respectively (Fig. 3c). Just to highlight a few examples, *Gm27606 (RLTR16C derived)* and *Timd2 (RLTR14-int)* were only accessible in the liver, but at different developmental stages; *1700020M21Rik (ORR1E)* only became accessible in the lung at P0; *Gm8113 (IAPEY4_LTR)* was accessible across all five tissues but in a developmentally dependent manner: early in intestine and lung, but late in liver and kidney (Fig. 3d; https://bit.ly/2EVYLjX).

Our previous study revealed a tight connection between activation of TEs and the binding of transcription factors [14, 57]. Based on motif enrichment analysis in the developmental stage-specific accessible TEs, we found that the TF binding motifs (TFBS) were stage-specifically enriched (Fig. 3e; Additional file 8: Table S3 to S7). To understand the expression pattern of these TFs in two distinct developmental stages, we further analyzed the corresponding RNA-seq data and examined the expression level of these TFs in all five tissues. As expected, we noticed a developmental stage-specific expression pattern of enriched TFs (Fig. 3f). For example, we found that the GATA binding motif was enriched in both intestine and liver at different developmental stages. Previous studies revealed the importance of *GATA4* and *GATA6* in the development of both intestine and liver [58–63]. In our study, we observed that *GATA5* and *GATA6*, but not *GATA4*, were upregulated in intestine development (Additional file 8: Table S8). Moreover, we found *GATA1* had the highest expression in the GATA family (~ 20 fold higher than *GATA4* and *GATA6*) in the liver at E14.5 and was most significantly downregulated at P0 stage (Additional file 8: Table S8). This result suggested that *GATA1* might also play important roles in regulating accessible TEs and contribute to liver development. We also found the motif of bZIP TFs, including *Jun*, *Junb*, *Batf*, and *Atf3*, was highly enriched in multiple tissues at P0. Correspondingly, the expression of these TFs was upregulated during embryo development (Additional file 8: Table S8).

### Accessible TEs altered the tissue specificity of gene expression

**Fig. 4:**
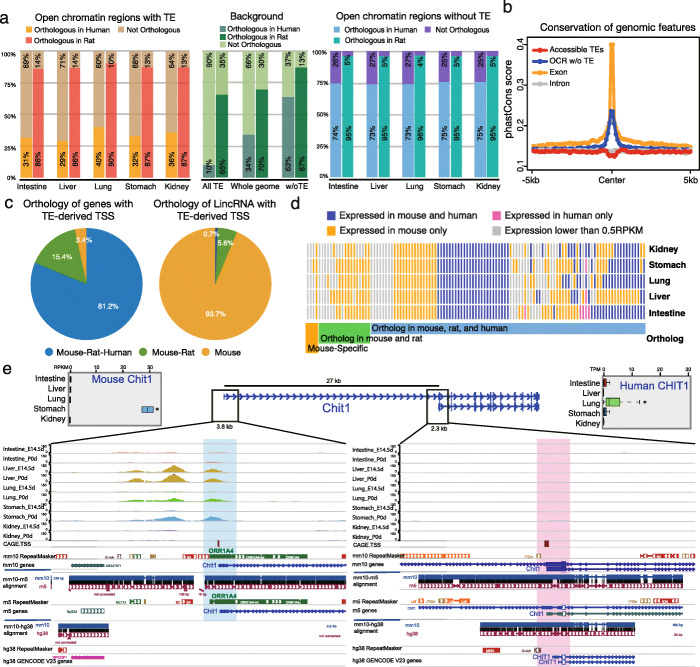
Evolutionary conservation of mouse accessible TEs in the rat and human genome. **a** The distinct proportion of orthologous mouse accessible TEs in the rat and human genome (left), orthologous mouse background regions in the rat and human genome (middle), and orthologous mouse OCRs without TEs in the rat and human genome (right). **b** Averaged phastCons score at 10-kb regions centered by accessible TEs (red), OCRs without TEs (blue), gene exon (orange), and intron (gray) in the mouse genome. Accessible TEs showed the lowest phastCons score in the center. **c** Distinct orthologous conservation of protein-coding genes (left) and lincRNA (right) with TE-derived TSS. In total, 87% of mouse protein-coding genes with TE-derived TSS were ortholog in both human and rat genome, while less than 1% of mouse lincRNAs were ortholog in other two species. **d** Expression pattern of 117 protein-coding genes with mouse TE-derived TSS in five tissues of mouse and human. **e** Epigenome browser view of ATAC-seq signals and genome-alignment (mm10, hg38, and rn5) around rodent-specific ORR1A4 TE-derived TSS (left: conserved in mouse and rat) and canonical ortholog TSS (right: conserved in mouse, rat, and human) of *Chit1* gene. Top-left: expression pattern of *Chit1* in five mouse tissues. Top-right: expression pattern of *CHIT1* in five human tissues. ANOVA test: *p*-value < 0.01

Next, we examined the evolutionary conservation of accessible TEs identified in our study. Based on current genome annotation and alignment [64], we found 10% of mouse TEs had orthologous counterparts in the human genome, and 65% had orthologous sequences in the rat genome. For accessible TEs, these two numbers were 30% and ~ 87% (Fig. 4a; Additional file 9: Table S1). In contrast, close to 75% of the non-TE open chromatin regions in the mouse genome had orthologs in the human genome, and 95% had orthologous sequences in the rat genome (Fig. 4a; Additional file 9: Table S1). These results supported a previous finding that regulatory elements, including TE-derived elements, were more likely to be retained in the genome [65]. By comparing the 60-ways PhastCons conservation of accessible TEs with that of their surrounding genomic sequence, we found that accessible TEs had much lower PhastCons scores than gene-coding exon, OCR without TEs, and intronic regions (Fig. 4b). These results suggested that most of the mouse accessible TEs were putative rodent-specific regulatory elements and were much less conserved compared to non-TE regulatory elements, at least based on sequence alignment-based estimates.

We further examined the conservation of accessible TEs that contained the 5′ end TSSs of genes (Fig. 4c; Additional file 9: Table S2). Consistent with overall accessible TEs, these TSS-deriving TEs were also mostly rodent-specific (59%). However, their downstream genes exhibited a more complex evolutionary pattern. Protein-coding genes were highly conserved: 81% of the 117 protein-coding genes with TE-derived TSSs had orthologs in all three species; only 18 did not have orthologs in the human genome, and only four were mouse-specific (Additional file 9: Table S3). In stark contrast, 94% of the 114 lincRNA genes with TE-derived TSSs were mouse-specific (Additional file 9: Table S4). This result was consistent with previous studies that suggested that species-specific TEs contributed to the origination of lincRNAs during evolution [7, 50, 66, 67].

Next, we asked how these domesticated TSS-deriving TEs influenced expression of their host genes. We compared the expression pattern of 95 conserved protein-coding genes across five tissues of mouse and human. We found more than half of these protein-coding genes showed distinct expression patterns at the tissue level between human and mouse (Fig. 4d). This result suggested that the domestication of TEs as a TSS may provide a mechanism to allow new tissue-specific gene expression to evolve. For example, *Chit1* encodes an enzyme called Chitinase 1, which is secreted by activated macrophages and plays a role in the degradation of chitin-containing pathogens [68, 69]. In the mouse genome, the 5′ TSS of *Chit1* is located in ORR1A4, which is a rodent-specific TE and not present in the human genome (Fig. 4e). This TE had strong accessibility in the mouse liver and stomach, but relative weak accessibility in the lung. Transcriptome analysis indicated *Chit1* was expressed the highest in mouse stomach (Fig. 4e; ANOVA test: *p-*value < 0.01; Additional file 5: Fig. S2). Interestingly, we did not detect *Chit1* expression in the RNA-seq data of mouse liver, possibly because the liver still missed certain cooperating transcription factors to initiate the transcription. Then, 27 kb downstream from this TE-derived TSS lied the canonical TSS, which is orthologous to the TSS of the human *CHIT1* gene. Interestingly, we did not observe any ATAC-seq signal around this conserved ortholog TSS in any of the five mouse tissues in our study (Fig. 4e), suggesting that this alternative TSS was not used in mouse genome for transcription initiation. In the corresponding five human tissues, *CHIT1* was only expressed in the lung, which contains a large population of alveolar macrophages [70]. We reasoned that the conserved TSS represented the ancestral element and was responsible for *CHIT1* expression in human lung tissue. However, the integration of ORR1A4 upstream of the canonical TSS in the rodent genome created a stronger TSS and a new first exon to initiate the transcription of *Chit1* in the stomach, potentially due to the sequence features of ORR1A4 that could respond to the stomach-specific transcription factors. Thus, a transposable element integration event not only resulted in the promoter turnover of nearby genes, but also initiated the gene transcription in a novel tissue.

### Domesticated TEs created tissue-specific expression of species-specific genes

**Fig. 5:**
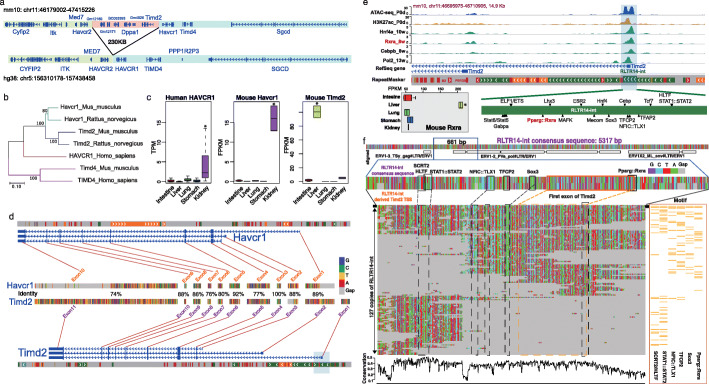
Ortholog analysis of *Timd2* gene. **a** Genome structure of *Havcr2-Timd2-Havcr1* in the mouse genome (top) and genome structure of *HAVCR2-HAVCR1* in the human genome (bottom). A 230-kb-long DNA fragment contained *Timd2* gene only exists in the mouse genome. **b** Gene ortholog analysis of *Havcr1*, *Timd2*, and *Timd4* in human, mouse, and rat based on protein sequence. *Havcr1* genes from mouse and rat were firstly clustered with *Timd2* genes instead of *HAVCR1* gene in the human genome. **c** Expression pattern of *Havcr1* and *Timd2* in five tissues of human (left) and mouse (middle and right). ANOVA test: *p* value < 0.01. **d** Pair-wise alignment of exons between *Havcr1* (top) and *Timd2* (bottom). RLTR14-int-derived Exon1 of *Timd2* did not have ortholog exon in *Havcr1*. **e** Top: Epigenome browser view of ATAC-seq and ChIP-seq signals around RLTR14-int-derived TSS of *Timd2* in mouse liver. The signals of ATAC-seq, H3k27ac, and TFs binding were co-localized at *Timd2* TSS region within RLTR14-int element. Bottom-left: expression pattern of *Rxra* in five mouse tissues. *Rxra* showed the highest expression in the liver. ANOVA test: *p-*value < 0.01. Bottom-right: predicted TF binding motifs within Timd2-TSS RLTR14-int element. **f** Pair-wise alignment among 128 RLTR14-int copies (overlapped to RLTR14-int-derived *Timd2* TSS) against to RLTR14-int consensus sequence. Top: Gene structure of full-length consensus sequence of RLTR14-int (5317 bp). Middle: 661 bp pair-wise alignment between consensus sequence and *Timd2*-TSS RLTR14-int element*.* Six TF binding motifs were identified at the exactly same positions in both consensus sequence and *Timd2*-TSS RLTR14-int element. Bottom-left: 661 bp pair-wise alignment and conservation of consensus sequence (1st row), *Timd2*-TSS RLTR14-int element (2nd row), and 127 RLTR14-int copies. Bottom-right: occupancy of six TFBSs matched to pair-wise alignment

About 19% of protein-coding genes that utilized an accessible TE-derived TSS were rodent- or mouse-specific (Fig. 4c). We noticed that 11 of these 22 rodent- or mouse-specific protein-coding genes were expressed in a tissue-specific manner (Fig. 4d). *Timd2* (T cell immunoglobulin and mucin domain-containing 2), a gene that can enhance T cell activation, was found to use an RLTR14-int element to initiate its liver-specific expression. *Timd2* is a rodent-specific gene, which originated from a genome duplication event in the mouse and rat genomes. A 230-kb-long DNA fragment containing *Timd2*, *Dppa1*, and several non-coding genes were duplicated and positioned inversely in the mouse genome between *Havcr2* and *Havcr1* (Fig. 5a). *Timd2* was a paralog of *Havcr1* resulting from the genome duplication. Both *Timd2* and *Havcr1* encode a 305aa protein with 60% identity at the amino acid level and 74% identity at the coding DNA level (Fig. 5b; Additional file 5: Fig. S3). *Havcr1* (Hepatitis A virus cellular receptor 1) is also called *KIM-1*(Kidney Injury Molecule 1), and is highly upregulated in injured kidneys [71]. *HAVCR1* belongs to TIM family, which also includes *HAVCR2* and *TIMD4*. As receptors for phosphatidylserine, TIM proteins bind many families of viruses such as hepatitis A, dengue, and ebola. We confirmed that *Havcr1* was highly expressed in normal kidney across human, mouse, and rat. However, *Timd2* was only expressed in mouse liver but not in the kidney (Fig. 5c; Additional file 5: Fig. S4; ANOVA test: *p-*value < 0.01).

A close examination of the pair-wise alignment between mouse *Timd2* and *Havcr1* revealed the homology between the two genes as well as a *Timd2*-specific first exon, which was derived from a rodent-specific RLTR14-int element (Fig. 5d; Additional file 10). Transcription factor binding motifs, including *Hnf4a*, *Cebp*, *Tfap2*, and *RxRa*, were predicted to be present in this RLTR14-int element, and the element was accessible and displayed strong RNA Polymerase II binding (Fig. 5e). ChIP-seq data also confirmed the binding of transcription factors, including *Hnf4a*, *Cebpb*, and *Rxra*, to the RLTR14-int (Fig. 5e). *Rxra* exhibited a strong liver-specific expression pattern, whereas *Hnf4a* and *Cebpb* expressions were more ubiquitous across all the tissues (ANOVA test: *p* value < 0.01). These results suggested that following the genomic duplication that created *Timd2*, the integration of RLTR14-int created an alternative promoter of *Timd2* that rewired its liver-specific expression pattern, likely by responding to liver-specific transcription factors such as *Rxra* (Fig. 5e).

Motif analysis of the RLTR14-int consensus sequence revealed six TF binding motifs (SCRT2/HLTF, STAT1::STAT2, NFIC::TLX1, TCF2, Sox3, Pparg::Rxra) in the region corresponding to new *Timd2* promoter derived from the RLTR14-int element (Fig. 5f). All six TFBSs were well conserved in the RLTR14-int element that gave rise to *Timd2* promoter. Alignment of 1451 RLTR14-int genomic fragments to the consensus sequence identified another 127 RLTR14-int copies that partially overlapped with the same region that derived *Timd2* promoter. However, none of the 127 copies contained the full set of six TFBSs identified in the consensus sequence (Fig. 5f), although some of them were accessible TEs in different tissues (Additional file 5: Fig. S5). These results suggested that the key TFBSs were probably already present in the ancestral RLTR14-int, and the element that produced *Timd2* promoter likely inherited the conserved TFBSs from the ancestral retrovirus.

**Fig. 6:**
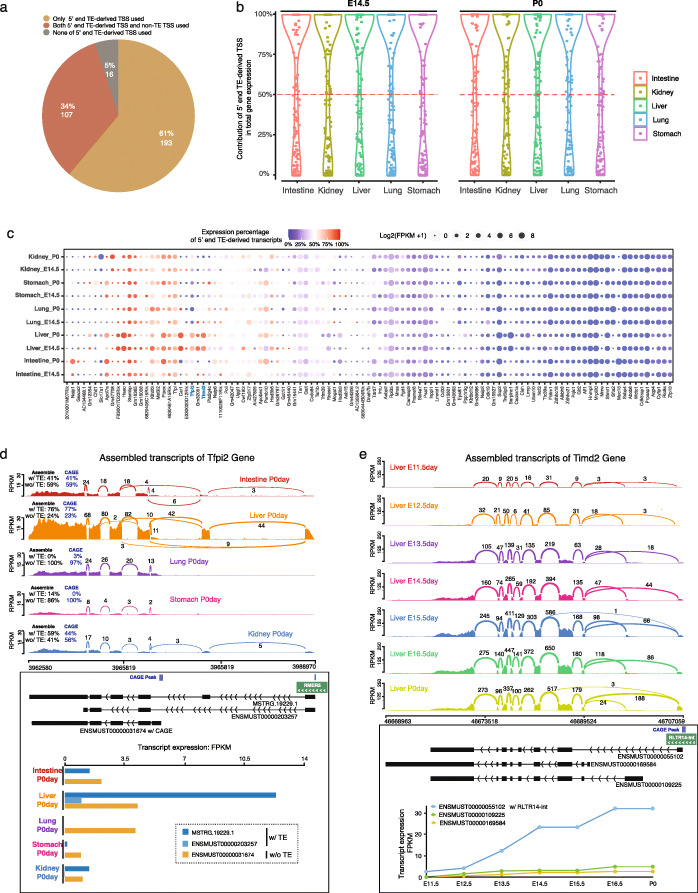
Contribution of 5′ end TE-derived TSS in tissue-specific gene expression. **a** Usage of 5′ end TE-derived TSS of 300 genes expressed in at least one tissue (> 0.1 FPKM). **b** Contribution of 5′ end TE-derived TSS in gene expression at two developmental stages in five mouse tissues. *Y*-axis: the expression percentage of 5′ end TE-derived transcripts contributed to the total gene’s expression at both E14.5 and P0 stage in each tissue. **c** Usage of 5′ end TE-derived TSS of 107 genes that used both 5′ end TE-derived and non-TE-derived TSS in five tissues at two developmental stages. The color of each dot represented the expression percentage of 5′ end TE-derived transcripts, and the size indicated the total gene expression values (FPKM). **d** Assembled transcripts of *Tfpi2* genes at the P0 stage in five mouse tissues. Top: Sashimi plot visualization of splicing-junctions from RMER5-derived 1^st^ exon to next exon in intestine, liver, and kidney. Bottom: expression of three assembled *Tfpi2* transcripts across five tissues. **e** Assembled transcripts of *Timd2* genes from E11.5 to P0 stages in mouse liver. Top: Sashimi-plot visualization of splicing-junctions from RLTR14-int-derived 1^st^ exon to next exon in all seven developmental stages. Bottom: expression of RLTR14-int-derived *Timd2* increased 13-fold across seven developmental stages in mouse liver

Having identified a substantial contribution of TEs to evolving new TSSs, we next systematically determined the usage of the TE-derived TSS in comparison to the usage of alternative, non-TE TSS of the same 453 genes. Towards this end, we performed full-length transcript assembly and calculated each transcript’s expression level in five tissues at two different developmental stages. Of the 453 genes with TE-derived TSS, 316 genes had detectable expression in at least one tissue (> 0.1 RPKM). In total, 193 (61%) of these genes used 5′ end TE-derived TSS exclusively to initiate transcription, e.g., *Cd302* and *Ms4a4d* (Additional file 5: Fig. S6A and S6B). A total of 107 (34%) of them utilized both 5′ end TE-derived TSS and non-TE TSS. Only 16 (5%) did not use 5′ end TE-derived TSS in these five tissues (Fig. 6a; Additional file 11: Table S1). We also assessed the contribution of 5′ end TE-derived TSS in producing transcripts and found that most genes had a higher fraction of their total transcripts originated from 5′ end TE-derived TSS across all five tissues and two developmental stages (Fig. 6b).

To further quantify the pattern of differential usage of 5′ end TE-derived promoter, we compared the expression level of transcription initiated from 5′ end TE-derived promoter with the non-TE-derived promoter for the 107 genes which utilized both types of promoters. A little more than half (55/107) of the genes used non-TE-derived promoters more often, and when they did so, the pattern was uniform across all five tissues. In contrast, the remaining half (52/107) used 5′ end TE-derived promoters more often, and when they did so, the pattern was tissue-specific (Fig. 6c). *Tfpi2*, a tumor suppressor gene and a member of Kunitz-type serine proteinase inhibitors [72], was found to use an RMER5-derived promoter to initiate 76% of gene transcription in the liver, but this promoter was not used in the lung and stomach at all (Fig. 6d). *Car1*, a carbonic anhydrase involving erythrocytes oxygen release, was specifically expressed in the developmental liver via a MER74C-derived promoter (Additional file 5: Fig. S6C). Finally, we observed that the usage of TE-derived promoters could be associated with developmental stages. *Naip1*, a NLR family gene encoding apoptosis inhibitor protein 1, was only transcribed in the P0day intestine by using an ORR1E-derived promoter (Additional file 5: Fig. S6D). The expression of *Timd2* in the liver increased more than 13 times from E11.5day to P0day, and the RLTR14-int derived promoter contributed almost exclusively to this increase (Fig. 6e).

To provide an orthogonal validation of discovery we made from the transcript assembly of RNA-seq data, we analyzed the CAGE data of five matching tissues generated by FANTOM5 [73]. In total, 114 of 453 candidate genes expressed in five mouse tissues during embryo development, and 75 of them had CAGE peaks at TE-derived promoters (Additional file 11: Table S2). By assessing the expression level of TE-derived promoter and non-TE-derived promoter for each gene using CAGE data, we found that about half of the genes only used the TE-derived promoter to initiate gene expression at both E14.5 and P0 (Additional file 5: Fig. S7A, S7B). Of the 18 genes with multiple TSSs, eight were predominantly using their TE-derived promoters to initiate gene expression based on CAGE signals (Additional file 5: Fig. S7C). Importantly, we found very high concordance between CAGE-seq signal and assembled RNA-seq expression in TE-derived promoter usage (Additional file 5: Fig. S7D).

## Discussion

Recent high-throughput sequencing technologies to characterize the transcriptomes and epigenomes of multicellular eukaryotes have revealed the crucial roles of TEs in the evolution of gene regulation. Integration of TEs into host genomes has brought numerous TF binding sites, and such perturbation of the genome interfered with gene regulatory networks and increased the regulatory dynamics of the genome when responding to external stimuli [18–23]. Specifically, the intact transcriptional elements of TEs can create novel transcription start sites (TSSs) to initiate transcription in the host genome. These novel TSSs were found to be the origination of a large proportion long non-coding RNAs [24, 46, 48, 50, 51, 66] and also created novel transcript isoforms of conserved protein-coding genes during evolution [8, 17, 20, 37, 74, 75].

Here we conducted a study to understand the functionality of TEs during mouse embryo development and focused on the activation of TE-derived TSSs in the development of five tissues. Leveraging the public datasets generated by ENCODE, we analyzed the epigenome data of five tissues (intestine, liver, lung, stomach, and kidney) at two different developmental stages: embryonic day 14.5 (E14.5) and postnatal day 0 (P0) (Additional file 1). On average, we observed that about 21% of the OCR in mouse development could be derived from TEs, suggesting TEs could escape from epigenetic silencing of the host genome during evolution. We noticed that liver had more accessible TEs, which account for 28% of the regulatory regions during development, when compared to the other four tissues (Fig. 1a). Such a phenomenon might be associated with the distinct evolutionary pressure and speed of gene regulatory network evolution in different tissues [76–78].

Regulatory elements, especially enhancers, are activated in a tissue-specific pattern [13–15, 28, 34, 79, 80]. In our study, we observed that accessible TEs showed highly tissue-specific patterns. Only ~ 10% of total accessible TEs were found to be accessible in all five tissues (Fig. 1c), and most of these constitutively accessible TEs were already accessible in mouse embryonic stem cells (Fig. 1d). Such results suggested that these TEs might be involved in very early-stage development and associated with fundamental biological processes, such as cell cycle regulation, and support findings of previous studies [14, 24, 28, 32, 33, 52, 53]. To explore the roles of TEs in tissue development, we examined the differential accessibility of TEs by comparing E14.5 and P0 development stages for each tissue. Interestingly, most accessible TEs in the stomach and kidney did not show differential accessibility between these stages; this might reflect different developmental timing between the mouse stomach, kidney, and other tissues. Conversely, in the intestine, lung, and liver, nearly half of accessible TEs showed distinct chromatin accessibility between the two development stages (Fig. 3a) and reflected a significant change of gene regulation at a later embryonic developmental stage. Previous studies indicate that transcription factors (TFs) play important roles in tissue development and are highly enriched in the open chromatin regions [16, 34, 56, 67, 81–83]. In our study, we identified 15 enriched TFBSs in the developmental stage-specific accessible TEs. We further observed the developmental-specific expression pattern of these TFs in the five tissues. Similar to previous studies [9, 34, 48, 52, 56, 82], our results indicated the importance of TEs in gene regulatory networks, especially in the formation of tissue specificity. Such results suggested that the accessible TEs were regulated by developmental stage-specific TFs, including *FOXA* and *NF1*, which have been reported to play essential roles in the development of the lung [84–87].

In addition to distal accessible elements that are far from gene promoters, we found about 10% of accessible TEs can serve as the transcription start site (TSS) of a gene. Our study identified 453 accessible TE-derived 5′ end TSSs across the five tissues, and most of these TE-derived TSSs were validated by independent CAGE-seq data generated by the FANTOM5 project [73, 88]. Previous studies reported that TEs were major contributors to the origin of non-coding RNAs [24, 46–51]. In addition to confirming TE’s contribution to lincRNA, our results revealed a crucial evolutionary mechanism by which TEs contributed significantly to evolving alternative transcription start sites for protein-coding genes and, in some cases, to evolving novel tissue-specific expression patterns for these genes. We found that 95 of 117 protein-coding genes with TE-derived TSSs had orthologous genes in the rat and human genome.

Interestingly, we found that most of these accessible TSS TEs were rodent or mouse-specific: 66 of the 95 conserved protein-coding genes used a rodent/mouse-specific TE-derived TSS to initiate transcription in different tissues (Additional file 9: Table S3). This result highlights that regulatory elements can evolve much more rapidly than genes themselves. By evolving a new TSS or a new promoter, a gene can rapidly explore a variety of expression patterns across cell types, and potentially offer phenotypic diversity upon which selection can act. Here, transposable elements provide convenient building blocks for the evolution of new promoters, and thus presenting a unique opportunity to accelerate regulatory evolution for the species that they invade. For example, a rodent-specific ORR1A4 initiates stomach-specific transcription of the *Chit1* gene. *Chit1* is secreted by activated macrophages and plays a role in the degradation of chitin-containing pathogens [68, 69]. The active expression of *Chit1* in the stomach might help the newborn mice to defend against pathogens encountered during feeding in early life. However, whether the expression of *Chit1* comes from macrophages or stomach cells, such as parietal cells or mucous neck cells, still needs further investigation via other experimental approaches, such as single-cell RNA sequencing analysis or in situ hybridization.

We also found that TEs can initiate the novel expression pattern of rodent-specific genes. *Timd2* is a rodent-specific gene that could enhance T cell activation by interacting with *SEMA4A*. *Timd2* is the duplicated gene of *Havcr1*, which is highly conserved and expressed in the kidney across mammals, including mouse, rat, and human. Conversely, the *Timd2* gene is only identified in the rodent genome and is especially highly expressed in the liver. Our analysis indicated that the insertion of the RLTR14-int element created a new 5′-end exon and initiated the transcription of *Timd2* in mouse liver by recruiting liver-specific TFs, which can be inferred by motif analysis and ChIP-seq assays (Fig. 5e). Another interesting finding is that the *Timd2* did not use the ancestral TSS that mainly initiates the expression of the *Havcr1* gene in the kidney. We noticed that two SINE elements, B1_Mm and B1_Mus2, inserted around the ancestral TSS of *Timd2*. Our motif analysis revealed that several repressive TF binding sites, including those of *SNAI2*, *FOXP1*, and *FOXD3*, were present in these two SINE elements (Additional file 5: Fig. S8). Previous studies indicated KRAB-ZFPs could repress the activity of LTRs through histone modification and DNA methylation, and further repressed the expression of nearby genes in both ES cells and adult tissues [89, 90]. Thus, it is tempting to hypothesize that the integration of the two SINE elements around the ancestral TSS of *Timd2* resulted in the silencing of that TSS, which indirectly facilitated the RLTR14-int-derived TSS to take over the expression of *Timd2*.

## Conclusions

TEs are significant contributors to genome evolution, and the percentage of TEs in the genome is positively correlated with the size of the genome [91]. Species-specific TEs are found to play roles as regulatory elements in human and mouse [5, 7, 18, 20, 34, 92]. Our study not only confirms previous findings, but also extends the contribution of TEs to the initiation of gene transcription, especially to creating novel tissue-specific gene expression patterns by acting as alternative TSSs. We additionally found that TEs may also silence genes through the repressive mechanisms of the host genome. Further investigation is required to confirm such a hypothesis. In summary, our study provides a comprehensive investigation of TEs’ function during mouse development. We report that TE-derived alternative TSSs of protein-coding genes can drive distinct tissue-specific expression patterns among different species during evolution and might eventually contribute to unique evolutionary advantages by increasing tissue plasticity.

## Methods

### Raw sequence data and processing

Raw fastq files of ATAC-seq and RNA-seq data for five mouse tissues were gathered from ENCODE data portal (https://www.encodeproject.org/), including intestine, liver, lung, stomach, and kidney (Additional file 1) [39–42]. Two different development stages were considered in each tissue: embryonic day 14.5 (E14.5) and postnatal day 0 (P0) [39, 40]. The alignment bam files and narrowPeak bed files of H3K27ac for these five tissues with two development stages were also downloaded from ENCODE (Additional file 1).

ATAC-seq data of five tissues with two development stages were separately processed by AIAP package that contained an optimized ATAC-seq data QC and analysis pipeline with default parameters [93]. Open chromatin regions (OCR) generated by AIAP were used in downstream analysis. Then, the two replicates of peaks of same tissue from two different stages were combined by using mergeBed [94].

RNA-seq data of five tissues with two development stages were processed by Cutadapt (v2.7; --quality-cutoff = 15,10 --minimum-length = 36), FastQC (v0.11.4), and STAR (v2.5.2b; --quantMode TranscriptomeSAM --outWigType bedGraph --outWigNorm RPM) to do the trimming, QC report, and mouse genome mapping (mm10) [95–97]. Then, the mouse gene expression was calculated by featureCounts (-p -T 4 -Q 10) [98] based on GENCODE vM20 gene annotation. The expression (TPM) of *CHIT1* and *HAVCR1* in those five human tissues were obtained from “GTEx_Analysis_2017-06-05_v8_RNASeQCv1.1.9_gene_tpm.gct” file, which was processed and normalized by GTEx Portal (https://www.gtexportal.org/home/) [99].

The H3K27ac alignment (bam file) and narrow peaks (bed file) of five mouse tissues at two developmental stages were downloaded from ENCODE data portal. The bamtobed of bedtools was used to process bam file and generate fragment file (bed files), which were further used to calculate the signal density (CPM) under each peak by using the intersectBed (-wa -c) command. The high confident H3K27ac narrow peak (with H3K27ac signal CPM > 3) were combined to the union peak set by using mergeBed, and used for downstream analysis.

### Transposable element annotation data and spatial correlation with peaks

The transposable elements (TEs) data of mouse (mm10) were obtained from the UCSC database (http://hgdownload.soe.ucsc.edu/goldenPath/mm10/database/) [100]. And the simple repeats, satellites, and TEs shorter than 100 bp were removed from this TE dataset.

The GenometriCorr (Genometric Correlation) is an R package that is used to calculate the spatial correlation of genome-wide interval datasets with the null hypothesis: interval sets are spatially independent [7, 43]. The correlated interval sets would follow a detectable pattern of the location, such as being consistently nearby or far away from each other in genomic coordinates, or preferentially overlapping. The permutation tests with 100 times were performed to look for TE enrichment in the open chromatin regions.

### Identification of accessible TEs

The accessible TEs in five mouse tissues were separately identified by two methods: (1) accessible TEs were firstly identified by the overlapping between TE and OCR peak with intersectBed (-f 0.5 -F 0.2), at least 50% overlap required as a fraction of TE element and at least 20% overlap required as a fraction of peak; (2) we calculated the Tn5 insertions of remaining TEs from method 1. Genome-wide Tn5 insertion counts were generated by AIAP, and Tn5 insertion of each remaining TEs and OCR peaks of five tissues at two development were calculated by using intersectBed, and further were normalized to the insertion per kilobase per million insertions (IPKM) based on total insertions of libraries and length of TEs and OCR peak. The 25% quantile IPKM of OCR peaks in each library was used as cutoff (Additional file 5: Fig. S9), and remaining TEs from method 1 with an IPKM over the cutoff were considered as accessible TEs. Finally, the accessible TEs identified by the above two methods were combined and used for analysis in the study. The coordinates of accessible TEs can be found in Additional file 2.

The intersectBed [94] was used to determine the numbers of accessible TEs across genomic features (exons, first exons, introns, and intergenic regions), which were defined by using GENCODE vM20 gene annotation, in the five mouse tissues. Then, the enrichment ratio of accessible TEs in each genomic feature was calculated by dividing the number of accessible TEs in each genomic feature to the number of all TEs in each genomic feature (Additional file 4: Table S2; Additional file 5: Fig. S1).

ATAC-seq data of mouse embryonic stem cell was obtained from GEO (accession number: GSE94249) and processed by the AIAP package to generate OCR peaks (ESC peaks) representing the chromatin open status in early development stage [101]. The accessible TEs identified in five mouse tissues that can overlap to ESC OCR peaks were defined as open in this early development stage (Additional file 4: Table S5).

### Subfamily enrichment of accessible TEs

To measure the subfamily enrichment of accessible TEs in each tissue, log enrichment ratio (LER) was calculated by the observed number of accessible TEs in the subfamily over the expected number of all TEs in the subfamily across open chromatin regions by the following formula:

$$ {\mathrm{LER}}_{ij}=\log 2\left(\ \frac{\mathrm{obsevered}\ \mathrm{Number}\ \mathrm{of}\ \mathrm{accessible}\ \mathrm{TE}\ i\ \mathrm{in}\ \mathrm{TE}\ \mathrm{subfamiliy}\ j}{\mathrm{Number}\ \mathrm{of}\ \mathrm{TE}\ i\ \mathrm{in}\ \mathrm{TE}\ \mathrm{subfamily}\ j\ast \frac{\mathrm{length}\ \mathrm{of}\ \mathrm{open}\ \mathrm{regions}\ \mathrm{withTE}}{\mathrm{length}\ \mathrm{of}\ \mathrm{genome}}}\ \right) $$

The individual copy numbers in each TE subfamily were count by using TE annotation dataset described above. The enriched subfamily of accessible TEs was calculated and filtered with the following two criteria: (1) the number of individual TE copies in one subfamily should be greater than 10, and (2) the LER value should be greater than 3 (Additional file 4: Table S7).

### GO analysis of tissue-specific accessible TEs

Accessible TEs that were only identified in one tissue was defined as tissue-specific accessible TEs. GO enrichment analysis of tissue-specific accessible TEs was performed by using GREAT (version 3.0.0) [102] with the following settings: (1) species assembly: mouse, NCBI build 38; (2) background regions: whole genome; (3) association rule: basal plus extension. Top 20 biological process terms were generated by GREAT with cutoffs of Binom FDR Q-Val < 0.05 and Hyper FDR Q-Val < 0.05 simultaneously (Additional file 6). GO enrichment analysis of accessible TEs constitutively identified in five tissues was also performed by using GREAT with the above settings.

### Defining genes associated with accessible TE-derived TSS

Mouse gene annotation (Gencode.vM20.annotation.gtf) was obtained from GENCODE to define the relationship between genes and accessible TEs [103]. The 500 bp upstream of full-length genes defined as the gene’s promoter region were used to overlap with accessible TEs by intersectBed. TE-derived TSS was defined by the intersection between accessible TEs and furthest 5′ end transcription start sites (TSS) of genes. The gene-type distribution of accessible TEs-derived TSS across five tissues was generated based on Gencode.vM20.annotation.gtf file of mouse genes (Additional file 7). CAGE-TSS data from FANTOM5 were used to validate accessible TEs-derived TSS across five tissues (Additional file 7) [73]. The examples of accessible TE-derived TSS and CAGE TSS locations were visualized on WashU Epigenome Browser [104].

### Differential accessible TEs and motif enrichment analysis

For each tissue, we calculated reads count table of peaks at E14.5 and P0 by using AIAP package and used edgeR to identify differential accessible regions (DARs) in the comparison between two development stages (abs (log2(Foldchange)) > 1 and FDR < 0.05, Additional file 5: FigureS10) [105, 106]. Then, the accessible TEs overlapped with DARs were identified as differential accessible TEs between two stages in five tissues: (1) those accessible TEs with positive foldchange were defined as P0day-specific TEs that were more open at P0; and (2) accessible TEs with negative foldchange were defined as E14.5day-specific TEs that were more open at E14.5. The rest of the accessible TEs did not show the difference of accessibility between two stages (Additional file 8: Table S1).

To explore the relation of differential accessible TEs on the gene expression at two stages, we assigned E14.5day-specific and P0day-specific TEs in five tissues to their nearest genes (the distance between TE and gene should be less than 20 kb), and the expression of those associated genes was tested by using the Wilcoxon signed-rank test to assess the significance of the difference between two development stages.

The enriched TFBS motifs of differential accessible TEs at E14.5 and P0 in five tissues were respectively discovered by using findMotifsGenome.pl (-size given) of HOMER software [107]. The enriched de novo motifs were selected with three conditions: (1) at least 10% of differential accessible TEs contained the motif; (2) match score of motifs should be greater than 0.9; (3) *P* value of the motif should be less than 1e−11. Then the known transcription factor genes (TF genes) with a motif match score > 0.9 to the enriched de novo motifs were extracted from HOMER results (Additional file 8).

### Estimating the evolutionary conservation of accessible TEs

The evolutionary conservation of accessible TEs was measured by using phastCons conservation scores from mm10.60way.phastCons.bw file downloaded from UCSC, which contained conservation scores for alignments of 59 vertebrate genomes with mouse genome generated by phastCons program [100, 108]. The bigwig file was transferred to the bedGraph file with bigWigToBedGraph tool [109]. In total, 20,000 accessible TEs were randomly chosen and expanded 5 kb from the center at both sides, and their base-pair level phastCons scores were obtained by using intersectBed. Then, each 10-kb region was divided into 100-bp windows, and the averaged conservation scores of each 100-bp region were computed separately. Finally, the conservation scores at 100 bp resolution of all 20,000 accessible TEs were averaged and plotted. The evolutionary conservation of different genomic features, including exons, introns, and peaks without TEs, was separately analyzed by using the above method with 20,000 10-kb regions that were randomly chosen from each genomic feature.

Open chromatin regions with TE or without TE in five mouse tissues were aligned to rat (rn6) and human (hg38) genome by using liftOver software with “-minMatch = 0.6” [64]. The mouse open chromatin regions that can successfully lift over to the rat or human genome were defined as orthologous in rat or human genome. Meanwhile, to measure the orthologous conservation at the genome-wide level, the whole mouse genome was first divided into 200-bp windows, and the orthologous regions were defined as the same standard as orthologous OCR described above, as well as those 200-bp windows that did not overlap with TEs. The orthologous conservation was also measured for the whole TEs in the mouse genome (Additional file 9: Table S1).

Ortholog protein-coding genes with TE-derived TSS in three species (mouse, rat, and human) were first determined by using ortholog information obtained from Ensembl [110]. The mouse-specific and mouse-rat ortholog genes were further validated by using blastp, the protein sequence of mouse genes against rat (taxid: 10116) and human (taxid: 9606) reference database on NCBI, with the cutoff of both coverage and identify > 60% [111].

To assess the conservation of lincRNA, the cDNA sequence of lincRNAs were obtained from “Long non-coding RNA gene annotation” file of GENCODE (vM21 version), and then blastn method with “somewhat similar sequences” parameter was used to search each cDNA against rat (taxid: 10116) and human (taxid: 9606) reference RNA sequences [103, 112]. The orthologous lincRNA was defined as both the identity and coverage of the top blast hit to rat, or human reference RNA sequences can be over 60% (Additional file 9).

### Conservation of the Timd2 gene

*Timd2* and *Havcr1* genes of mouse were identified as duplicated genes in the Duplicated Genes Database (DGD) [113]. The sequence of cDNA and protein between *Timd2* and *Havcr1* was obtained from the Ensembl database (https://useast.ensembl.org/Mus_musculus), and the pair-wise alignment results were generated by Mview (Additional file 5: Fig. S3) [110, 114]. The gene cDNA sequences of *Havcr1*, *Timd2*, and *Timd4* in mouse and rat were downloaded from the Ensembl database, multiple sequence alignment was performed by using muscle [115]. Then MEGA was used to construct the evolutionary tree of those genes with the neighbor-joining method [116].

The exon sequences of mouse *Havcr1* and *Timd2* genes were downloaded from the Ensembl database [117]. Exons of *Havcr1* were aligned to the *Timd2* sequence by using blastn with “somewhat similar sequences” parameter to identify the homologous exons between two genes [112]. The exon sequence coverage, identity, and gaps between homologous of *Havcr1* and *Timd2* were estimated based on the blastn results (Additional file 10).

FIMO software was used to identify the TFBS motifs in the RLTR14-int elements based on motif weigh matrix file (JASPAR_CORE_2016_vertebrates.meme) from JASPAR [118, 119]. The ChIP-seq signals of *Hnf4a*, *Rxra*, *Cebpb*, and *Pol2* genes were collected from GEO (GSM2406338, GSM1163178, GSM1854433, and GSM864688) and displayed on WashU Epigenome Browser [101, 104, 120].

The consensus sequence of RLTR14-int was downloaded from Repbase and was used to perform the pair-wise alignment with all RLTR14-int individual copies in the mouse genome by using muscle [115, 121]. The genomic information of proteins within the RLTR14-int consensus sequence was obtained from Dfam database [122]. FIMO software was applied to identify TFBS motifs for each RLTR14-int copy that can align to *Timd2*-TSS RLTR14-int element [119]. The base-pair conservation score of RLTR14-int alignments was calculated by the following formula:

$$ \mathrm{conservation}\ \mathrm{score}=\frac{\max \left(\mathrm{number}\ \mathrm{of}\ \mathrm{nucleotide}:\mathrm{one}\ \mathrm{ofA},\mathrm{T},\mathrm{C},\mathrm{G}\right)}{\mathrm{total}\ \mathrm{number}\ \mathrm{of}\ \mathrm{nucleotide}} $$

### Transcripts assemble

The mouse gene transcripts of five tissues at two development stages were assembled based on RNA-seq data. First, the sorted BAM files were generated after quality trimming by Cutadapt (v2.7) with parameter “-quality-cutoff = 15,10 --minimum-length = 36” and reads mapping to mouse genome (mm10) by STAR (version 2.5.2b) with the parameter “--outSAMtype BAM SortedByCoordinate --outFilterMultimapNmax 20 --outSAMattributes NH HI NM MD AS XS --twopassMode Basic --outFilterType BySJout --limitBAMsortRAM 100000000000” [95, 97]. Then, StringTie (v2.0.4) with the parameter “-f 0.15 -a 20 -j 10 -m 200” was applied to assemble transcripts based on sorted BAM files [123]. Next, StringTie took as the input of a list of transcripts GTF files with coverage of at least 10 and merged these transcripts into a non-redundant set and compared assembled transepts to GENCODE gene annotation vM20. The quantification of the unified set of transcripts was calculated by RSEM (v1.3.0; -p 12 --bam) separately for five tissues at two stages [124].

In total, 316 TE-derived TSS genes were found to express in at least one tissue (mean expression FPKM > 0.1) and were classified into three groups based on the TSS usage of expressed transcripts: (1) only 5′ end TE-derived TSS used; (2) none of 5′ end TE-derived TSS used; and (3) both 5′ end TE-derived TSS and non-TE TSS used (Additional file 11: Table S1). Then, the expression percentage of TE-derived transcripts in each gene was calculated by the sum of the expression of TE-derived transcripts dividing the total gene expression. Based on the RNA-seq alignment (bam files) and gene GFF annotation file, the sashimi plots of each gene were generated by using sashimi_plot command in MISO framework to visualize the RNA-seq read densities along with exons and junctions, as well as visualize the structure of the gene’s isoforms [125, 126].

CAGE-seq peak-based expression table of robust CAGE-seq peaks for mouse samples with annotation was downloaded from FANTOM5 project [73]. The expression table of CAGE peaks at the embryo development stage of the intestine, liver, lung, stomach, and kidney tissues was downloaded from the FANTOM data portal (https://fantom.gsc.riken.jp/data/) and further extracted by in-house script. The CAGE-seq peaks were assigned to either TE-derived TSS or non-TE TSS by using intersectBed with 1 bp overlap. Then the contribution of TE-derived TSS was calculated based on the expression level of CAGE-seq peak associated with TE-derived TSS in the total expression level of CAGE-seq peaks associated with both TE-derived TSS and non-TE TSS.

## Supplementary information

**Additional file 1.** The epigenomic data of five tissues at embryonic day 14.5 and postnatal day 0 development stages from the ENCODE.

**Additional file 2.** The accessible TEs identified in the five tissues.

**Additional file 3.** The spatial correlation between open chromatin regions and transposable elements (TEs) in five tissues.

**Additional file 4: Table S1.** The total number of peaks, H3k27ac regions, and accessible TEs in five tissues. **Table S2.** The genomic distribution of accessible TEs in 5 mouse tissues. **Table S3.** The number of accessible TEs shared among the five tissues. **Table S4.** Enriched TF binding motifs identified in TEs commonly accessible in five tissues. **Table S5.** The accessibility of TEs in mouse embryonic stem cells. **Table S6.** The class distribution of accessible TEs in five tissues. **Table S7.** The subfamily enrichment of those accessible TEs.

**Additional file 5: Fig. S1.** Genomic distribution of accessible TEs of 5 mouse tissues in the mouse genome. **Fig. S2.** Epigenome browser view of ATAC-seq and RNA-seq signals of *Chit1* gene. **Fig. S3.** Pair-wise alignment of cDNA and protein sequences between *Timd2* and *Havcr1* genes. **Fig. S4.** Epigenome browser view of ATAC-seq and RNA-seq signals of *Timd2* gene. **Fig. S5.** Motif analysis of RLTR14-int elements. **Fig. S6.** Sashimi plot for the transcripts of genes at two development stages of five tissues. **Fig. S7.** The TE-derived TSS genes with transcript start site overlapped with CAGE Peaks. **Fig. S8.** Pair-wise alignment between the DNA sequences around the TSS of mouse *Timd2* and *Havcr1* genes. **Fig. S9.** The distribution of Tn5 insertion number in TE and Peak at E14.5 and P0 development stages of 5 tissues. **Fig. S10.** Percentage of dynamically changed Peaks between E14.5 and P0 in five mouse tissues.

**Additional file 6.** The enriched GO biology process items of accessible TEs that only identified in one tissue.

**Additional file 7: Table S1.** The number of accessible TEs located in the gene’s promoter and contributed to the TSS of genes. **Table S2.** The annotation of accessible TE that derived TSS of genes in five tissue. **Table S3.** Examples of accessible TEs that derived TSS.

**Additional file 8: Table S1.** The dynamic changes of accessible TEs between two development stages of five tissues. **Table S2.** The number of stage-specific accessible TEs located in the gene’s promoter regions. **Table S3-S7.** The enriched TF binding motifs of stage-specific accessible TEs in intestine, liver, lung, stomach, and kidney. **Table S8.** Expression of TF genes at two development stages of five tissues.

**Additional file 9: Table S1.** The evolutionary conservation of open chromatin regions with TEs or without TEs across five mouse tissues by comparing with the human and rat genome. **Table S2.** Conservation of accessible TEs that contained the TSSs of genes in mouse, rat, and human. **Table S3.** The ortholog of accessible TE-derived TSS genes in mouse, rat, and human. **Table S4.** The ortholog of accessible TE-derived lincRNAs in three species.

**Additional file 10. **The percentage of identity, coverage, and gaps for the exon’s alignment between *Timd2* and *Havcr1* genes.

**Additional file 11: Table S1.** The number of genes that their transcripts used 5′ end TE-derived TSS and non-TE derived TSS. **Table S2.** The usage of TE-derived TSS in the transcripts of genes overlapped CAGE peaks.

**Additional file 12.** Review history.

## Data Availability

The ATAC-seq, RNA-seq, and H3k27ac data for 5 mouse tissues that were processed and analyzed in current study are available from ENCODE data portal, and their accession numbers were recorded in Additional file 1 [41, 42]. The CAGE data are available from FANTOM5 project [73]. ATAC-seq data of mouse embryonic stem cell is available from GEO (GSE94249) [101]. The ChIP-seq datasets are available from GEO (GSM2406338, GSM1163178, GSM1854433, and GSM864688) [101].
