# Supplementary material for: Tissue-specific usage of transposable element-derived promoters in mouse development
Source: Genome Biol. 2020 Sep 28;21:255. doi: 10.1186/s13059-020-02164-3 (PMC7520981; doi:10.1186/s13059-020-02164-3)
Supplement: Supplementary file 5 — Additional file 5: Fig. S1. Genomic distribution of accessible TEs of 5 mouse tissues in the mouse genome. Fig. S2. Epigenome browser view of ATAC-seq and RNA-seq signals of Chit1 gene. Fig. S3. Pair-wise alignment of cDNA and protein sequences between Timd2 and Havcr1 genes. Fig. S4. Epigenome browser view of ATAC-seq and RNA-seq signals of Timd2 gene. Fig. S5. Motif analysis of RLTR14-int elements. Fig. S6. Sashimi plot for the transcripts of genes at two development stages of five tissues. Fig. S7. The TE-derived TSS genes with transcript start site overlapped with CAGE Peaks. Fig. S8. Pair-wise alignment between the DNA sequences around the TSS of mouse Timd2 and Havcr1 genes. Fig. S9. The distribution of Tn5 insertion number in TE and Peak at E14.5 and P0 development stages of 5 tissues. Fig. S10. Percentage of dynamically changed Peaks between E14.5 and P0 in five mouse tissues. [file 13059_2020_2164_MOESM5_ESM.docx]

Supplementary figures:

Figure S1. Genomic distribution of accessible TEs of five mouse tissues in the mouse genome. Large number of accessible TEs located in the intragenic or intergenic regions of mouse genome.


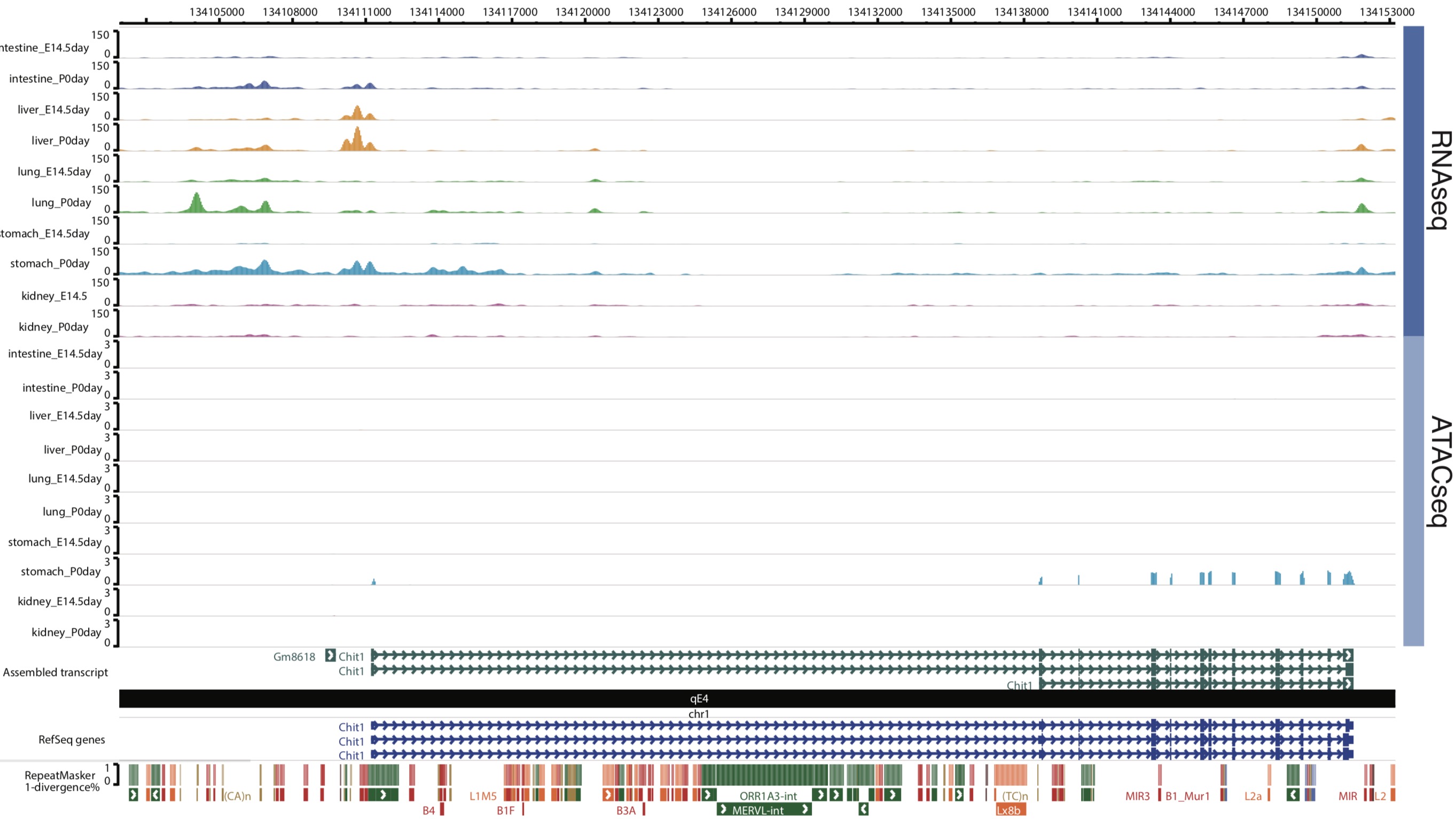


Figure S2. Epigenome browser view of ATAC-seq and RNA-seq signals of *Chit1* gene.


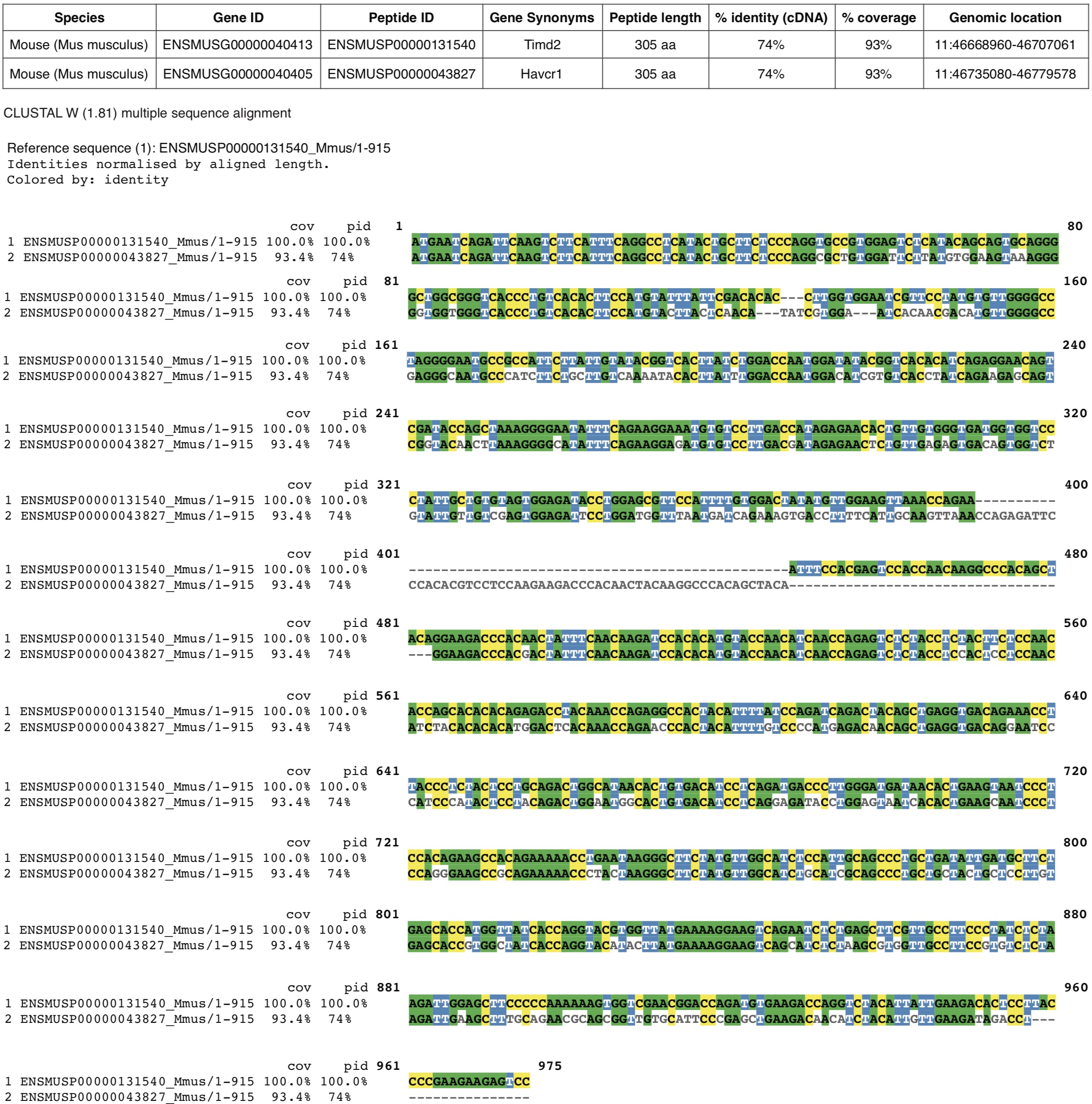


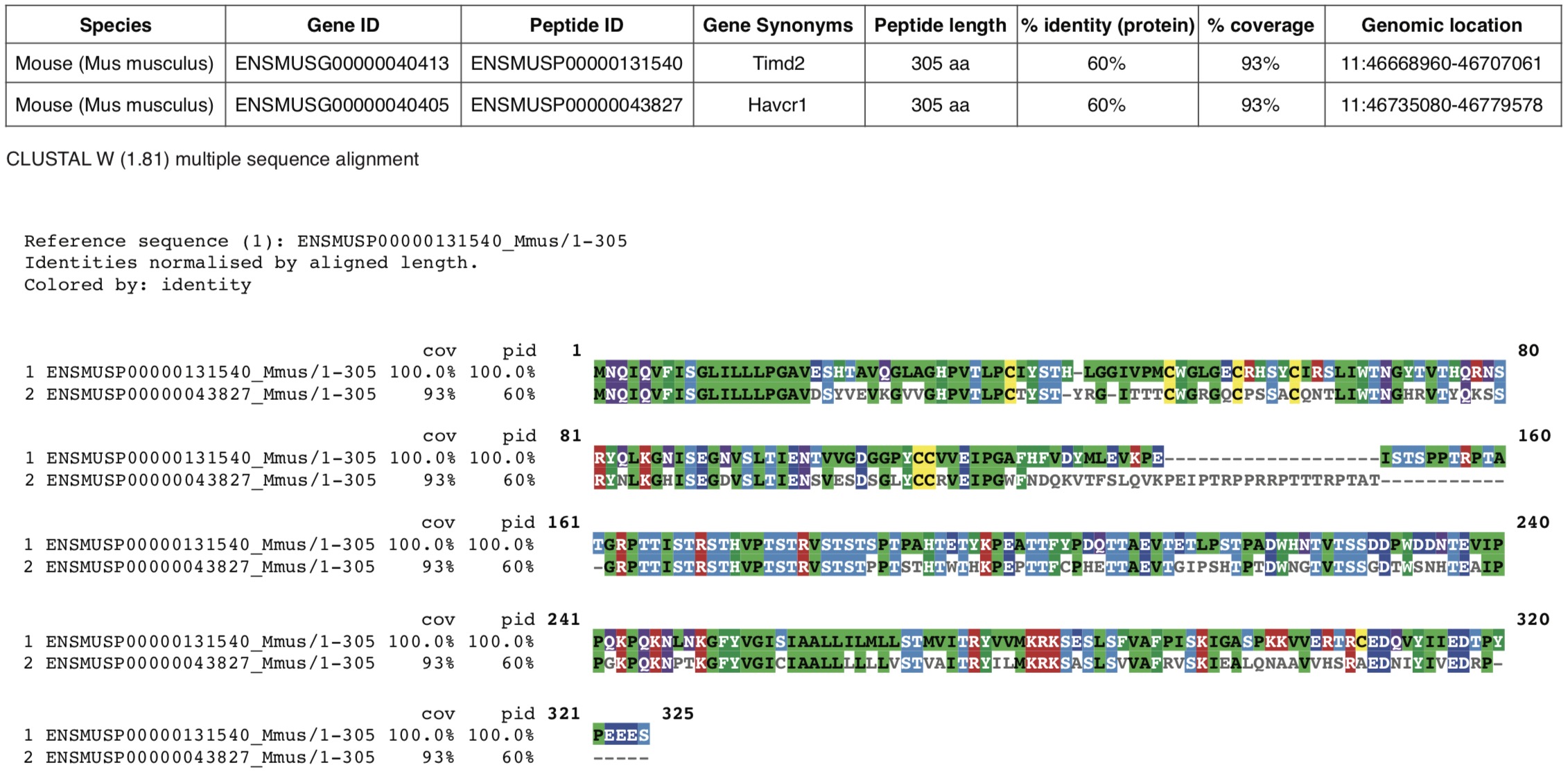


Figure S3. Pairwise alignment of cDNA and protein sequences between *Timd2* and *Havcr1* genes of mouse.


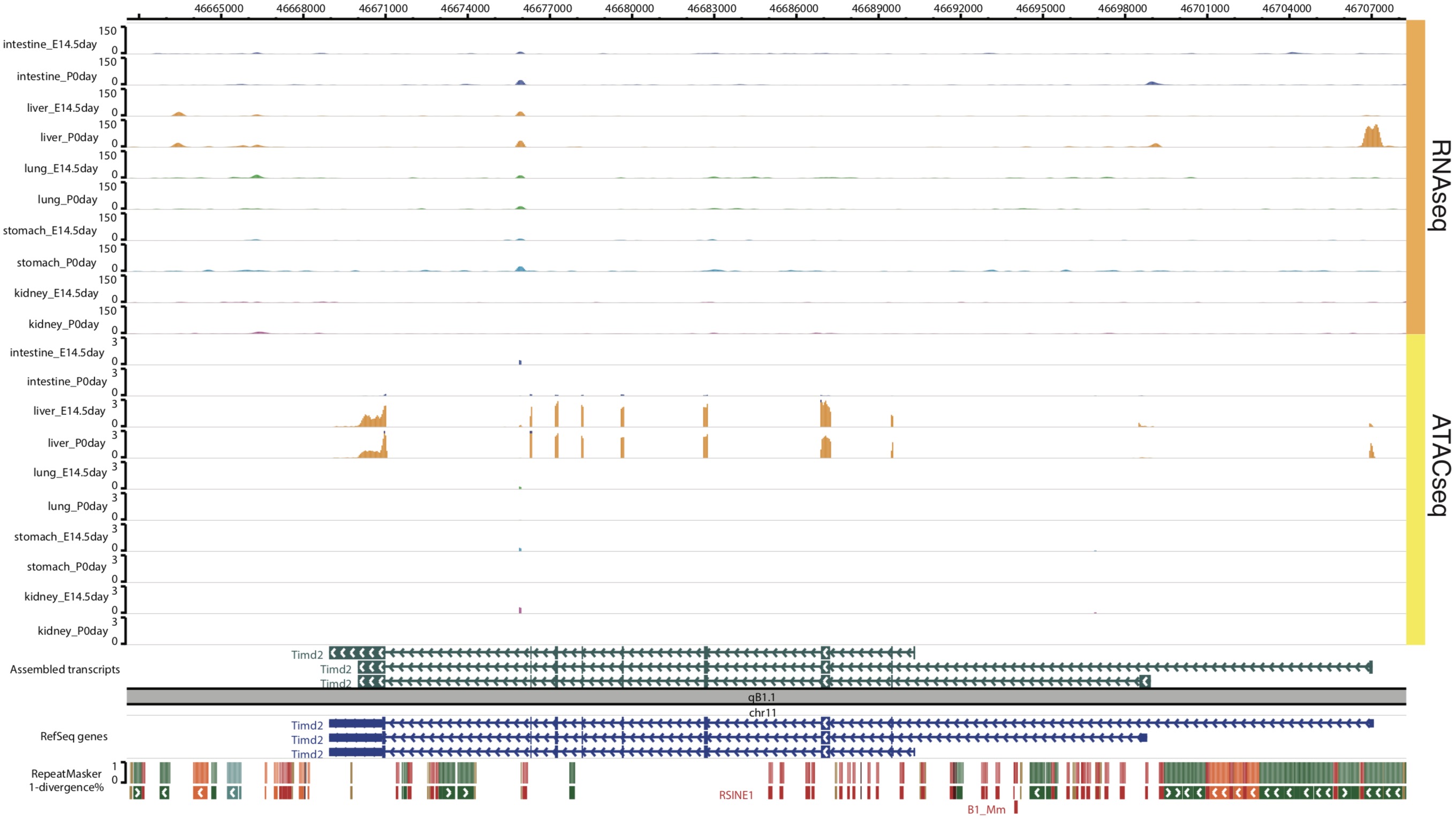


Figure S4. Epigenome browser view of ATAC-seq and RNA-seq signals of *Timd2* gene.


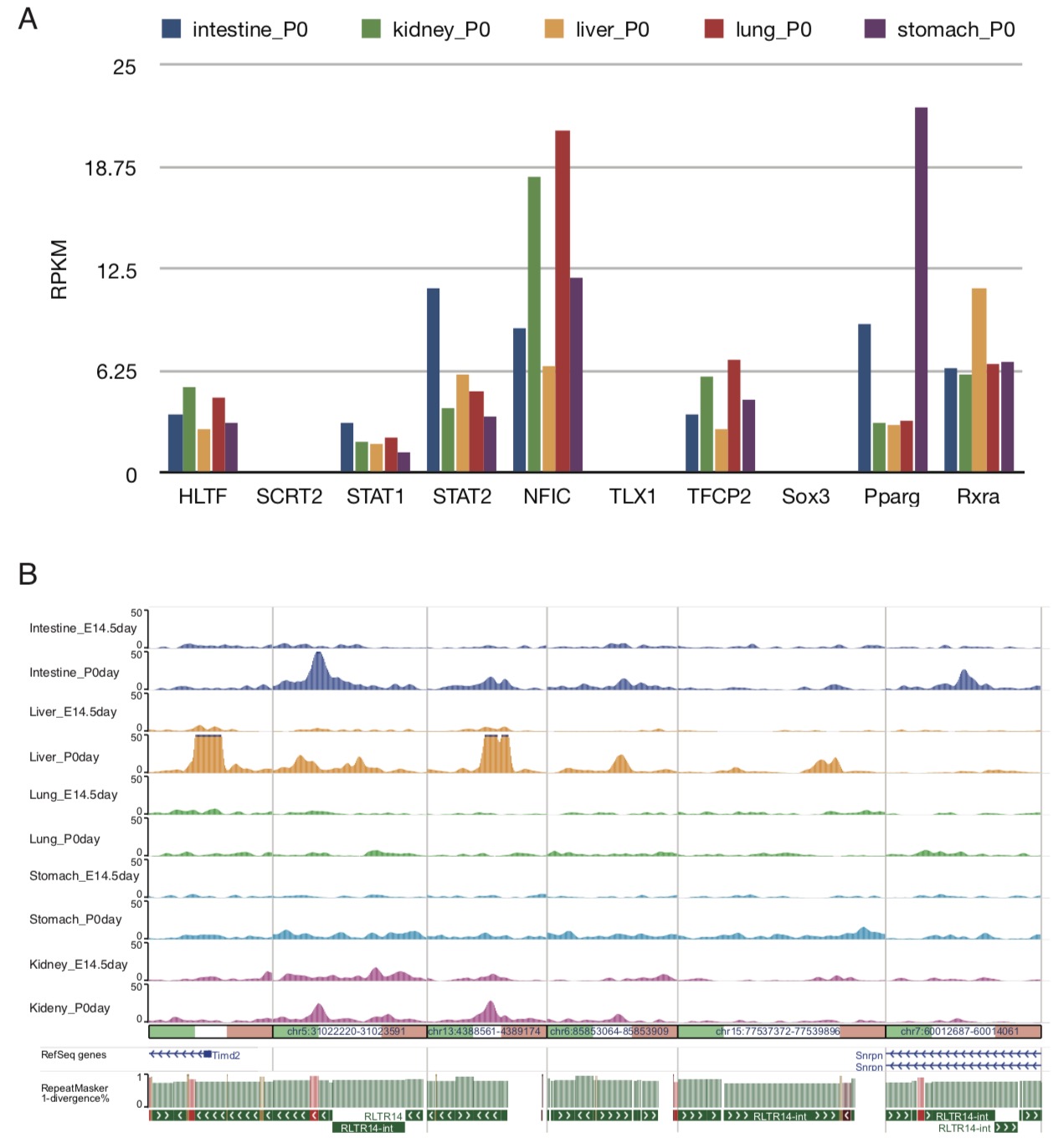


Figure S5. Motif analysis of RLTR14-int elements. **A.** The expression of TF genes in five tissues at P0 stage that were shared between RLTR14-int consensus sequence and RLTR14-int derived TSS of *Timd2* gene. **B.** Epigenome browser view of RLTR14-int elements that showed high ATAC-seq signals.


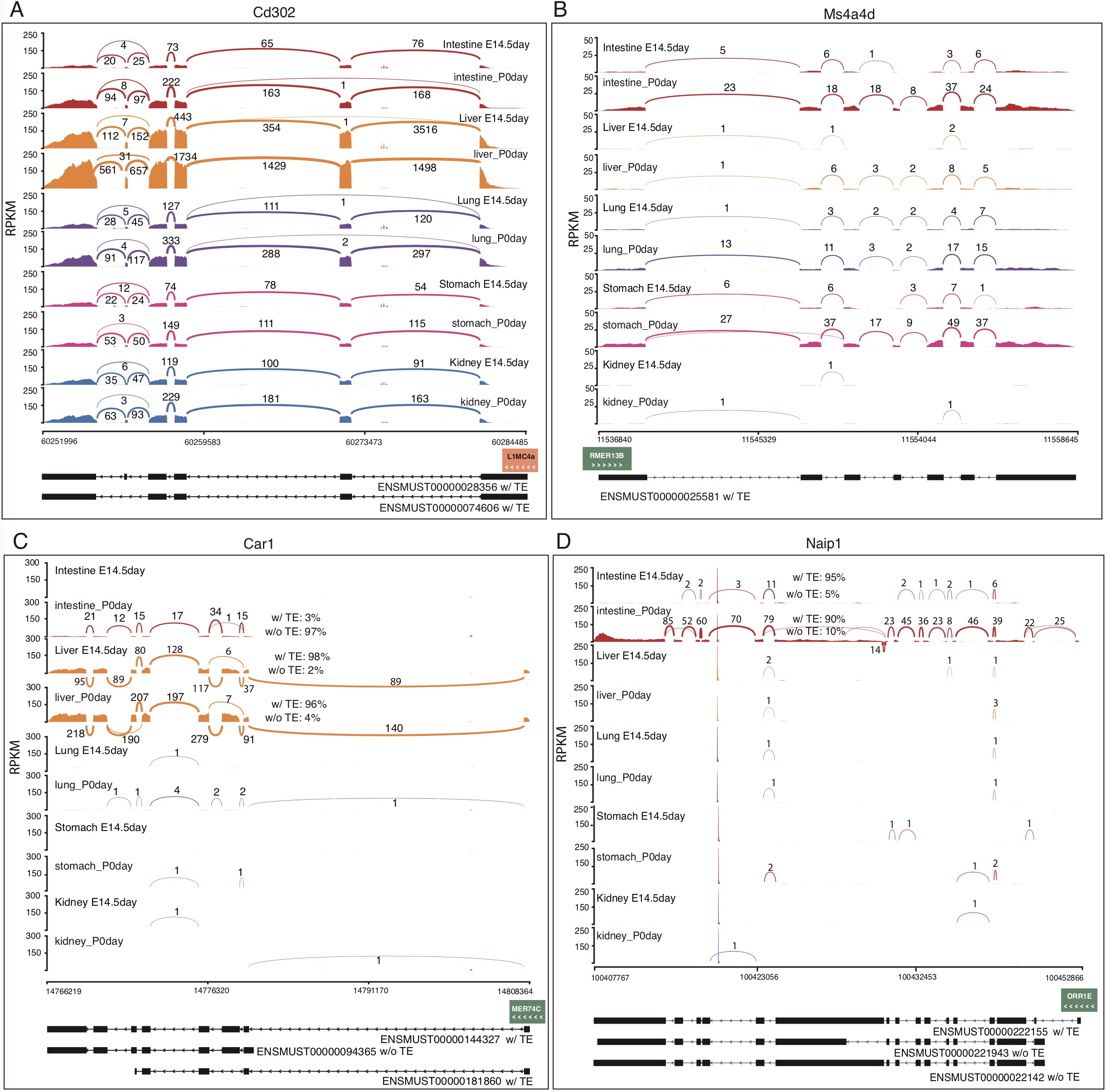


Figure S6. Sashimi plot for the transcripts of genes at two development stages of five tissues. The transcript starts sites of *Cd302* (**A**) and *Ms4a4d* (**B**) gene all derived from TEs. **C.** The *Car1* gene showed higher expression percentage of TE-derived transcripts in liver, but very lower percentage in intestine. **D.** The *Naip1* gene only showed high expression percentage of TE-derived transcripts in intestine.


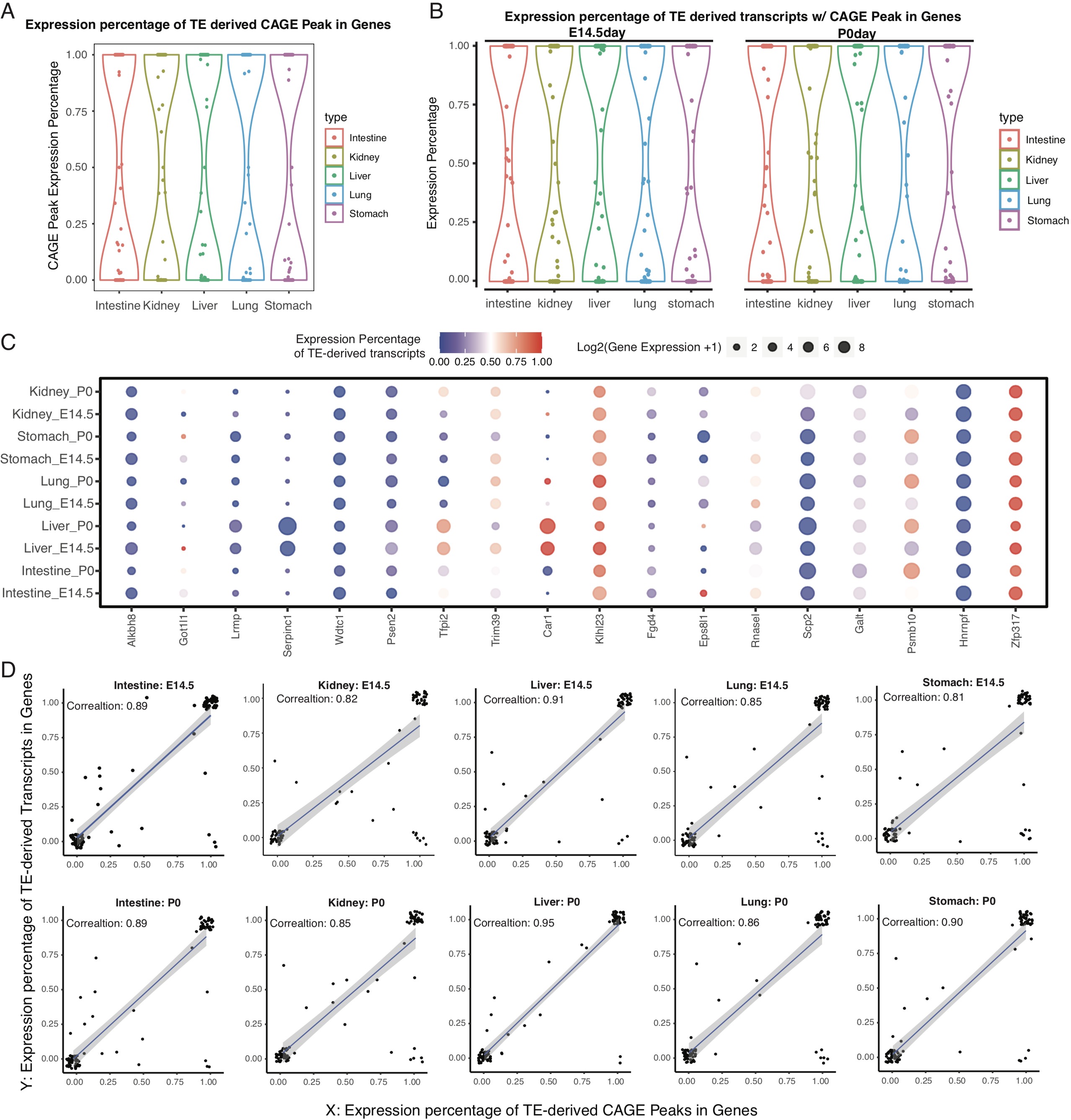


Figure S7. The TE-derived TSS genes with transcript start site overlapped with CAGE Peaks. **A.** The expression percentage of TE-derived CAGE peaks in the genes. **B.** The expression percentage of TE-derived transcripts that TSS overlapped with CAGE peaks at two development stages in five tissues. **C.** Bubble plot of genes with TE-derived and non-TE transcripts that TSS overlapped with CAGE peaks. **D.** The correlation of expression percentage between TE-derived CAGE peaks and TE-derived transcripts in genes across five tissues with different stages.


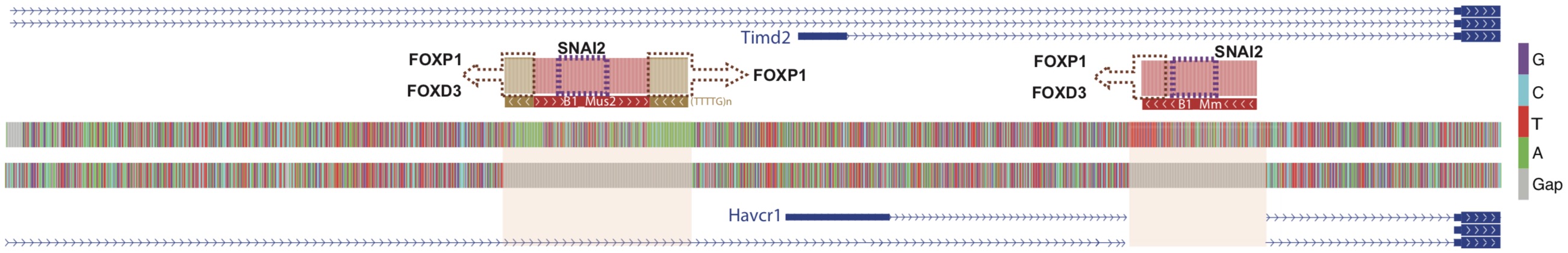


Figure S8. Pairwise alignment between the DNA sequences around the TSS of *Timd2* and *Havcr1* genes. There were two SINE elements inserted around the orthologous TSS of *Timd2* having several repressive TF binding sites.


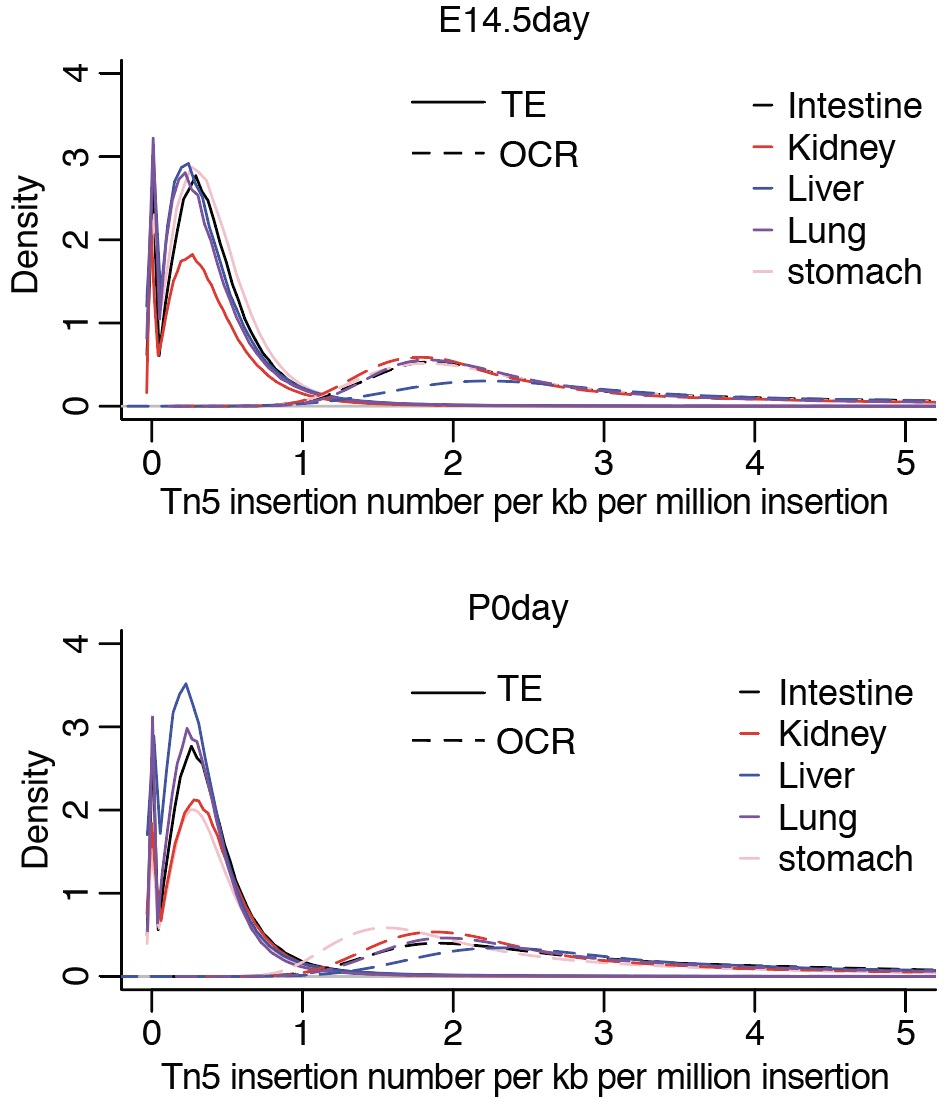


Figure S9. The distribution of Tn5 insertion number in TE and OCR at E14.5 and P0 development stages of 5 tissues. OCR: open chromatin region.


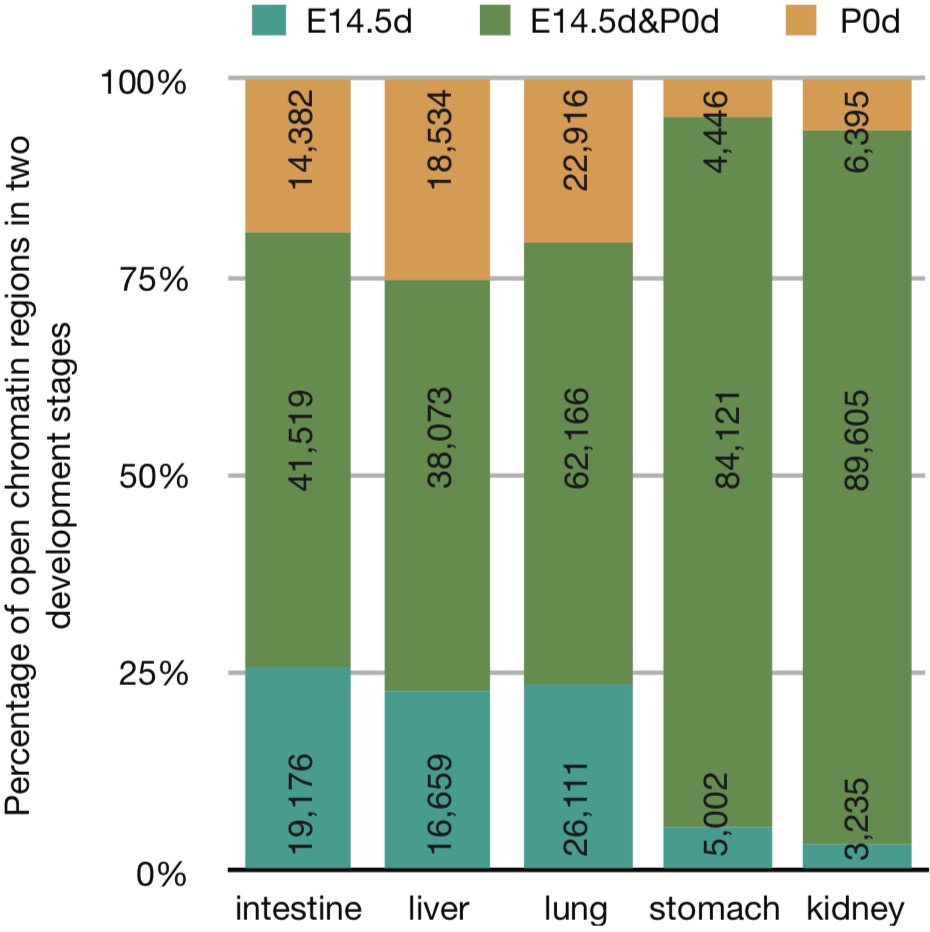


Figure S10. Percentage of dynamically changed Peaks between E14.5 and P0 in five mouse tissues. The peaks showed similar pattern of dynamic changes like the accessible TEs. About 50% of peaks showed changes of accessibility between the two developmental stages in intestine, liver and lung. But only about 10% of Peak showed changes in the stomach and kidney between E14.5 and P0.
