# Supplementary material for: Tissue-specific usage of transposable element-derived promoters in mouse development
Source: Genome Biol. 2020 Sep 28;21:255. doi: 10.1186/s13059-020-02164-3 (PMC7520981; doi:10.1186/s13059-020-02164-3)
Supplement: Supplementary file 12 — Additional file 12. Review history. [file 13059_2020_2164_MOESM12_ESM.docx]

Review History

**First round of review**

**Reviewer 1**

**Are you able to assess all statistics in the manuscript, including the appropriateness of statistical tests used?**

Yes, and I have assessed the statistics in my report.

**Comments to author:**

A rapidly growing number of studies have reported roles for transposable elements (TEs) in regulating host genes as enhancers or promoters. The genome-wide studies typically rely on datasets of epigenetic state, transcription factor binding, RNA-seq, etc. to identify potential roles for TEs in gene regulation. In this manuscript, the authors mine datasets on accessible chromatin in 5 mouse tissues from two developmental stages. They report on TEs in open/accessible chromatin, some of which are tissue-specific or developmental stage specific and ~450 of which create transcription start sites (TSSs) for protein coding genes or lncRNAs. They conclude that "TEs insertions increased the regulatory potential of the genome, and some TEs have been domesticated to become a crucial component of gene regulatory network during mouse tissue development."

The technical aspects of the study appear to be well done and the data is clearly presented. This paper is a solid contribution that adds to the literature on regulatory roles of TEs, although it does not provide much novel insight or break new ground. Moreover, to support the broad conclusions of the paper, functional studies need to be performed.

The following specific issues need to be addressed:

1.  In several places, the manuscript would benefit from additional information to put the results in better context.  For example, regarding evolutionary conservation (page 8-9), the authors found that 70% of accessible TEs did not have an ortholog in the human genome.  Is this different than the percentage for all TEs?  When giving such percentages throughout the paper, it would be helpful to compare to the "background" of all TEs.  For comparison, please add graphs with information on all TEs to Figures 1b and figure 4.  Please discuss any differences to the background of all TEs in the appropriate sections.

2. One of the main findings emphasized in the study is the use of TEs as tissue-specific promoters for host genes. In the conclusion, they state: "Our study not only validates previous findings, but also indicates the contribution of TEs in the initiation of gene transcription, especially to create novel tissue-specific expression patterns of protein-coding genes during evolution by acting as alternative TSSs." This finding is not novel, with a number of reports showing that TEs can be tissue-specific promoters, for example in early development (see Franke et al Genome Research 27:1384-1394; and other papers) and in placenta. This excellent review discusses this topic in great detail: "Long terminal repeats: from parasitic elements to building blocks of the transcriptional regulatory repertoire" by Thompson et al in Mol Cell. 2016 Jun 2; 62(5): 766-776. Notably, this review devotes a section to "LTRs as tissue-specific genic promoters". The authors need to make a better case for the novelty of this present study.

3. Related to the above, there have been several studies like this present one that use extensive data mining of epigenetic states, correlations with gene expression patterns, etc. to examine functionality of TEs. The current standard to prove functionality is to delete or genetically modify the TE and examine the effects. To justify the bold title and conclusions of the paper, such experiments need to be performed for at least a few selected cases.

4. The example of a TE as a transcription start site (TSS) most highlighted in the paper is an RLTR14-int element that provides a promoter for liver-specific expression of the mouse Timd2 gene (fig. 5). The authors present compelling evidence for this, including the binding of TFs to this TE. It should be noted, however, that the "int" designation of an LTR element refers to the interior portion of the retroviral-like sequence that typically does not naturally contain transcriptional regulatory motifs, normally found in the LTR portion. While such "int" regions certainly can occasionally act as promoters, this is presumably by "accident" or random chance. Do the authors think that an Int portion of an LTR element has any greater probability to become a promoter compared to any random piece of DNA? Does the consensus sequence of RLTR14-int contain those TF binding sites? This concept should be discussed and these statements at the bottom of page 11 (and similar statements elsewhere) modified or expanded:

"Integration of TEs into host genomes has brought numerous TF binding sites, and such perturbation of the genome interfered with gene regulatory networks and increased the regulatory dynamics of the genome when responding to external stimuli [18-20]. Specifically, the intact transcriptional elements of TEs to create novel transcription start sites (TSSs) to initiate transcription in the host genome."

5. The authors use the term "transposable elements" throughout the paper but it seems nearly all the enriched families are LTR elements, even though these are less numerous than LINEs and SINEs in the genome. Perhaps this fact is worth a comment.

6. For the examples of TE-derived TSSs highlighted in the figures (eg. Figure 2D), please give the genomic coordinates of the TE. Can the authors estimate the actual percentage contribution of the TE promoter to overall expression the gene (by using CAGE or RNA-seq and comparing to other promoters for the gene)? This would greatly help to gauge significance. For example, the first example shown (RNAse1), the MER90 (which this reviewer could not locate) is probably an alternative promoter, which is very minor in importance (judged by a quick look at the Genome Browser), but for the next case of NAIP2, it seems the ORR1E is the main promoter. Also, if any of the cases highlighted in the paper have been reported before, that should be acknowledged (such as for NAIP2, reported by reference 20 in the authors' reference list).

7. In the last results section (top of page 15), the authors comment that two SINE elements around the TSS of Timd2 have binding motifs for repressive TFs, which cause them to hypothesize that TEs can also carry repressive TF binding sites to silence gene expression. This concept is not novel as other examples of TEs being transcriptional repressors have been published. For example, see the work of Didier Trono.

8. Some of the figure legends are too brief and need more explanatory detail.

**Reviewer 2**

**Are you able to assess all statistics in the manuscript, including the appropriateness of statistical tests used?**

Yes, and I have assessed the statistics in my report.

**Comments to author:**

In their manuscript "Transposable elements shape tissue-specific gene expression during mouse development", the authors use mouse ENCODE ATAC-seq and RNA-seq data to investigate TE accessibility, and their impact on gene expression across 4 different tissues. They found that TEs were associated with over 20% of accessible chromatin regions during development, with both tissue-specific and developmental stage-specific variability. They suggest that many of these TEs were co-opted as functional gene promoters, rewiring developmental gene expression in a tissue-specific manner, based on the transcription factor binding sites found within the TE sequences.

The scientific question they try to address is interesting and compelling. However the way in which the analyses were conducted is not rigorous enough to support all of the findings. Not a single p-value is shown in the entire manuscript, since apparently, they did not conduct statistical tests to support most of their findings. In particular, ~40% of the mouse genome is made of TE, so I would expect that about 1 out of 2 accessible regions would overlap a TE by chance.

For example, in Line 115 the authors write: "…. In each tissue, TEs were associated with 17% (kidney) to 28% (liver) of accessible regions, and about half of these accessible TEs directly overlapped with the active histone modification H3K27ac…". Are those percentages more than expected by chance? How many accessible (or acetylated) regions are found overall in the kidney and in the liver? Would the difference in numbers of accessible regions across tissues affect their conclusions?

There are dozens of similar statements in the manuscript, not fully supported by statistics.

In the last few years, there were many papers investigating TE recruitment in gene regulatory networks and most of them were based on enrichment analyses rather than overlap analyses. In other words, given a set of accessible regions, how many of them are statistically enriched with TEs? As a matter of fact, there is a computational tool designed for this scope (PMID: 23637635), which was used in most of the above mentioned papers. This tool performs permutation tests (or binomial if preferred) to look for TE enrichment in a given set of genomics region.

To be clear, I completely understand that the main question is "how many TEs are accessible in each tissue at each time point?". And you do not need a statistical test to answer to such question. But without statistics the comparisons between tissues and between developmental stages are not supported.

The second major issue I found while reading the manuscript is that the methods are not described in a rigorous way. The authors mention the softwares used for each step of the analysis, but they never include any parameter For example in line 393 the sentence: "During the processing of RNA-seq data, we used Cutadapt, FastQC, and STAR to do the trimming, QC report, and genome mapping processes [74-76]." is lacking details.

Even worse, the sentence: "We also got the expression of some human genes in the 5 tissues from NCBI gene database" is not up to Genome Biology standard (or to any scientific publishing standard to be honest)

Minor comments:

- Line 138: Did the authors perform a motif analysis for TEs constitutively accessible to understand what is determining the widespread accessibility of these TEs across tissues?

- Line 163: Does developmental stage changes in TE accessibility correspond to change in expression of the associated gene? The authors should be able to address this using the RNA-seq data they have downloaded.

- Did the authors perform any comparison of their liver findings with the findings of a recent study the found significant TE recruitment in the primate liver gene regulation (PMID: 28855262)? Did they observe any conserved TE with a function in the liver gene regulation of both mouse and primates?

**Authors Response**

**Point-by-point responses to the reviewers’ comments:**

**We are particularly grateful for the reviewers’ enthusiasm for the quality of our work. We appreciate their constructive and thoughtful comments. Guided by reviewers’ suggestions, we performed additional analyses, and based on newly generated results, we believe we have addressed all of the raised concerns.**

**We have marked the edited texts in the original manuscript: the new text was marked with light-green.**

**Below we provide point-by-point responses with light-blue. The reviewer’s comments were marked with underline. The edited manuscript was cited in the response as “edited manuscript” in green.**

**Reviewer #1:** A rapidly growing number of studies have reported roles for transposable elements (TEs) in regulating host genes as enhancers or promoters.  The genome-wide studies typically rely on datasets of epigenetic state, transcription factor binding, RNA-seq, etc. to identify potential roles for TEs in gene regulation.  In this manuscript, the authors mine datasets on accessible chromatin in 5 mouse tissues from two developmental stages.  They report on TEs in open/accessible chromatin, some of which are tissue-specific or developmental stage specific and ~450 of which create transcription start sites (TSSs) for protein coding genes or lncRNAs.  They conclude that "TEs insertions increased the regulatory potential of the genome, and some TEs have been domesticated to become a crucial component of gene regulatory network during mouse tissue development."

The technical aspects of the study appear to be well done and the data is clearly presented.  This paper is a solid contribution that adds to the literature on regulatory roles of TEs, although it does not provide much novel insight or break new ground.  Moreover, to support the broad conclusions of the paper, functional studies need to be performed.

The following specific issues need to be addressed:

1.  In several places, the manuscript would benefit from additional information to put the results in better context.  For example, regarding evolutionary conservation (page 8-9), the authors found that 70% of accessible TEs did not have an ortholog in the human genome.  Is this different than the percentage for all TEs?  When giving such percentages throughout the paper, it would be helpful to compare to the "background" of all TEs.  For comparison, please add graphs with information on all TEs to Figures 1b and figure 4.  Please discuss any differences to the background of all TEs in the appropriate sections.

**Response**: We thank the reviewer for an important suggestion. Following this suggestion, we have provided more comprehensive background estimates to facilitate readers a better understanding of the likelihood of finding an ortholog for an accessible TE. The background now includes TE overall, non-TE genomic sequence, and genome overall. These new results are now in new Figure 4A and are provided below for the reviewer’s reference. Briefly, 10% and 65% of total TEs in the mouse genome have orthologs in human and rat, whereas 31% of accessible TEs have orthologs in human, and 86% have orthologs in the rat. These two parameters are 34% and 70% for general genomic regions, and 75% and 95% for the non-TE genomic sequence. We also used the background to calculate the enrichment of accessible TE in different genomic features, and updated Figure 1b. We have updated our manuscript to reflect these new analyses:

“We noticed that ~6% of accessible TEs overlapped with exons, and were especially enriched in the first exons comparing with the background (Fig. 1b). Such results suggested the potential role of TEs as promoters in mouse tissue development as previously reported [7, 19-24, 46-51]"

“In contrast, close to 75% of the non-TE open chromatin regions in the mouse genome had orthologs in the human genome, and 95% had orthologous sequences in the rat genome (Fig. 4a; Additional file 9: Table S1). These results supported previous founding that regulatory elements, including TE-derived elements, were more likely to be retained in the genome [65]”

**Fig. 4 Evolutionary conservation of mouse accessible TEs in the rat and human genome. a).** The distinct proportion of orthologous mouse accessible TEs in the rat and human genome (**left**), orthologous mouse background regions in the rat and human genome (**middle**), and orthologous mouse OCRs without TEs in the rat and human genome (**right**).

**Fig. 1** **Accessible transposable elements (TEs) in five mouse tissues.** **b).** Genomic distribution enrichment of accessible TEs of five mouse tissues in the mouse genome. Accessible TEs highly enriched in the exons of the mouse genome, especially the first exon.

2.  One of the main findings emphasized in the study is the use of TEs as tissue-specific promoters for host genes.  In the conclusion, they state: "Our study not only validates previous findings, but also indicates the contribution of TEs in the initiation of gene transcription, especially to create novel tissue-specific expression patterns of protein-coding genes during evolution by acting as alternative TSSs."  This finding is not novel, with a number of reports showing that TEs can be tissue-specific promoters, for example in early development (see Franke et al Genome Research 27:1384-1394; and other papers) and in placenta.  This excellent review discusses this topic in great detail: "Long terminal repeats: from parasitic elements to building blocks of the transcriptional regulatory repertoire" by Thompson et al in Mol Cell. 2016 Jun 2; 62(5): 766-776. Notably, this review devotes a section to "LTRs as tissue-specific genic promoters".   The authors need to make a better case for the novelty of this present study.

**Response:** We apologize for the less than scholarly writing of our manuscript. By no means that we wish to claim that we were the first to report a proof-of-concept that TE can become tissue-specific promoters, and we thank the reviewer for the criticism and for bringing critical literatures to our attention. We have thoroughly edited our language to make it more scholarly, including citing and discussing the studies mentioned by the reviewer. We do hope that our study substantially expanded previous studies by contributing numerous novel analyses and discoveries, in a manner that was almost predicted by the Thompson et al. review [1], and we carried out these analyses. Our analysis was the first to include comprehensive ATAC-seq mapping across multiple cell types and developmental stages; in addition to providing interesting and novel anecdotes, we provided a comprehensive analysis that allowed assessment of the genome-wide contribution of TEs; we also dissected the events of how new promoters can evolve in general, and how tissue-specific promoters can evolve; we determined different promoter usage by using both RNA-seq and CAGE-seq data to increase rigor. We hope the reviewer agrees that our comprehensive and rigorous analyses provided valuable and critical additions to the understanding of TE’s functional involvement in the evolution of gene regulatory networks, an exciting and rapidly moving field.

3. Related to the above, there have been several studies like this present one that use extensive data mining of epigenetic states, correlations with gene expression patterns, etc. to examine functionality of TEs.  The current standard to prove functionality is to delete or genetically modify the TE and examine the effects.  To justify the bold title and conclusions of the paper, such experiments need to be performed for at least a few selected cases.

**Response**: We completely agree with the reviewer that experimental validation is essential to rigorously validate our conclusions. We apologize for not including the desired experimental validation but hope the reviewer would consider our justification and the additional analyses we included in the revision.

First, the paradigm of using correlative genomics/epigenomics patterns to examine the functionality of TEs in several previous studies mostly aimed at predicting regulatory elements in TEs, for example, novel promoters or enhancers. Experimental validation helped to strengthen the key predictions. The TE-derived promoters we characterized in our study were already annotated as promoters by GENCODE, and our analysis revealed their novel evolutionary origin and tissue specificity. Thus, the confidence that they are bona fide promoters is high.

Second, most published experimental validations via genetically engineering TEs used cell-line based assays. A similar strategy for validating TE-derived tissue-specific alternative promoters in early mouse development is a low-throughput and lengthy process. The current Covid-19 pandemics unfortunately significantly impacted our lab’s ability to carry out wet-lab experiments. We are committed to following up on our interesting candidates, but certainly do not wish to delay publishing our genomics analysis by a year or two, the typical time to conduct the requested assays. We hope that the reviewer would allow us to rapidly disseminate our analysis results based on high-quality genomic data, and to pursue candidate driven analysis in future, independent studies.

Third, we now provide additional, in-depth analysis together with independent datasets to further improve the rigor of our findings. For example, we have applied reference-based transcriptome assembly to construct complete transcripts and used very stringent cutoff to reduce false-positive rate; we compared RNA-seq assembly based results with results from analyzing CAGE-seq data, an independent data resource from FANTOM5 project, and reported high concordance between these results; we systematically investigated the pattern of alternative promoter usage, including non-TE promoters and TE-derived promoters. These new results provide stronger support for the validity of our discovery on TE-derived tissue-specific and developmental stage-specific promoters. We have included these new results in two new figures, Fig 6 and Supplementary Fig 9, which we also inserted below for the reviewer’s reference.

Fourth, we agree that we should avoid making bold claims without comprehensive and rigorous experimental validation. Thus, we have carefully edited our manuscript to remove such statements, and modified our manuscript title to “Tissue-specific usage of transposable elements derived promoters in mouse development”.

  Fig. 6a, b, and c

**Fig. 6 Contribution of 5’ end TE-derived TSS in tissue-specific gene expression. a).** Usage of 5’ end TE-derived TSS of 300 genes expressed in at least one tissue (>0.1 FPKM). **b).** Contribution of 5’ end TE-derived TSS in gene expression at two developmental stages in five mouse tissues. Y-axis: the expression percentage of 5’ end TE-derived transcripts contributed to the total gene's expression at both E14.5 and P0 stage in each tissue. **c).** Usage of 5’ end TE-derived TSS of 107 genes that used both 5’ end TE-derived and non-TE-derived TSS in five tissues at two developmental stages. The color of each dot represented the expression percentage of 5’ end TE-derived transcripts, and the size indicated the total gene expression values (FPKM).

**Figure S7.** **The TE-derived TSS genes with transcript start site overlapped with CAGE Peaks**. **D).** The correlation of expression percentage between TE-derived CAGE peaks and TE-derived transcripts in genes across five tissues with different stages.

4. The example of a TE as a transcription start site (TSS) most highlighted in the paper is an RLTR14-int element that provides a promoter for liver-specific expression of the mouse Timd2 gene (fig. 5). The authors present compelling evidence for this, including the binding of TFs to this TE.  It should be noted, however, that the "int" designation of an LTR element refers to the interior portion of the retroviral-like sequence that typically does not naturally contain transcriptional regulatory motifs, normally found in the LTR portion.  While such "int" regions certainly can occasionally act as promoters, this is presumably by "accident" or random chance.  Do the authors think that an Int portion of an LTR element has any greater probability to become a promoter compared to any random piece of DNA?  Does the consensus sequence of RLTR14-int contain those TF binding sites?  This concept should be discussed and these statements at the bottom of page 11 (and similar statements elsewhere) modified or expanded: "Integration of TEs into host genomes has brought numerous TF binding sites, and such perturbation of the genome interfered with gene regulatory networks and increased the regulatory dynamics of the genome when responding to external stimuli[18-20]. Specifically, the intact transcriptional elements of TEs to create novel transcription start sites (TSSs) to initiate transcription in the host genome."

**Response**: We thank the reviewer for the keen observation and insightful comment. Indeed, the first exon of *Timd2* was derived from the PVa_pol region of an RLTR14-int element. As the reviewer pointed out, regulatory elements are often found within LTR elements, rather than the internal sequences of an endogenous retrovirus. Thus, we believe RLTR14-int represents a rare case, or “accidental event”, in which the internal sequences happened to carry relevant transcription factor binding sites and eventually exapted to become a new first-exon of the *Timd2* gene. Following the reviewer’s suggestion, we carefully analyzed the consensus sequence of RLTR14-int and other genomic fragments of this TE subfamily. Briefly, as we have shown, the *Timd2*-TSS RLTR14-int copy contained motifs of 6 TFs, including *SCRT2/HLTF*, *STAT1/STAT2*, *NFIC::TLX1*, *TFCP2*, *SOX3*, and *Pparg::Rxra*. Interestingly, the consensus sequence of RLTR14-int also contained these motifs. We found 127 other RLTR14-int genomic fragments that also contained the region from which the first exon of Timd2 derived. However, the same motif analysis indicated that none of them contained the full set of six motifs. Consistent with this result, none of these 127 fragments overlapped with any annotated TSSs, even though 5 of them exhibited accessible chromatin in our datasets. We now included this new analysis in a new figure 5f and S6B.

We updated our result and discussion as:

“Motif analysis of the RLTR14-int consensus sequence revealed six TF binding motifs (SCRT2/HLTF, STAT1::STAT2, NFIC::TLX1, TCF2, Sox3, Pparg::Rxra) in the region corresponding to new *Timd2* promoter derived from RLTR14-int element (Fig. 5f). All six TFBSs were well conserved in the RLTR14-int element that gave rise to *Timd2* promoter. Alignment of 1451 RLTR14-int genomic fragments to the consensus sequence identified another 127 RLTR14-int copies that partially overlapped with the same region that derived *Timd2* promoter. However, none of the 127 copies contained the full set of six TFBSs identified in consensus sequence (Fig. 5f), although some of them were accessible TEs in different tissues (Additional file 5: Figure S5). These results suggested that the key TFBSs were probably already present in the ancestral RLTR14-int, and the element that produced *Timd2* promoter likely inherited the conserved TFBSs from the ancestral retrovirus. “

**Fig. 5 Ortholog analysis of *Timd2* gene. f).** Pair-wise alignment among 128 RLTR14-int copies (overlapped to RLTR14-int derived *Timd2* TSS) against to RLTR14-int consensus sequence. **Top**: Gene structure of full-length consensus sequence of RLTR14-int (5317bp). **Middle**: 661bp pair-wise alignment between consensus sequence and *Timd2*-TSS RLTR14-int element*.* Six TF binding motifs were identified at the exactly same positions in both consensus sequence and *Timd2*-TSS RLTR14-int element. **Bottom-left**: 661bp pair-wise alignment and conservation of consensus sequence (1^st^ row), *Timd2*-TSS RLTR14-int element (2^nd^ row), and 127 RLTR14-int copies. **Bottom-right**: occupancy of six TFBSs matched to pair-wise alignment.

**Figure S5. Motif analysis of RLTR14-int elements. B).** Epigenome browser view of RLTR14-int elements that showed high ATAC-seq signals.

5. The authors use the term "transposable elements" throughout the paper but it seems nearly all the enriched families are LTR elements, even though these are less numerous than LINEs and SINEs in the genome.  Perhaps this fact is worth a comment.

**Response**: We thank the reviewer for the suggestion. In the revised manuscript, we now present a full analysis of accessible TEs with respect to their classification. Briefly, we now report that close to half of the accessible TEs were derived from SINE elements, and 11-16% were derived from LINE elements. LTR subfamilies indeed were more represented in the enrichment-based analysis. These new results are now in the new figure Fig 1f. We updated our result section and discussion as:

“Close to half of the accessible TEs belonged to the SINE class, and ~30% were derived from LTR elements. Of the remaining, 15% were LINEs, and less than 8% of accessible TEs belonged to DNA class (Fig. 1f; Additional file 4: Table S6). However, subfamily enrichment analysis of accessible TEs indicated that most of the top enriched TE subfamilies were LTRs (Fig. 1g; Additional file 4: Table S7).”

**Fig. 1 Accessible transposable elements (TEs) in five mouse tissues. f).** TE class distribution of accessible TEs in five mouse tissues.

6.  For the examples of TE-derived TSSs highlighted in the figures (eg. Figure 2D), please give the genomic coordinates of the TE.  Can the authors estimate the actual percentage contribution of the TE promoter to overall expression the gene (by using CAGE or RNA-seq and comparing to other promoters for the gene)?  This would greatly help to gauge significance. For example, the first example shown (RNAse1), the MER90 (which this reviewer could not locate) is probably an alternative promoter, which is very minor in importance (judged by a quick look at the Genome Browser), but for the next case of NAIP2, it seems the ORR1E is the main promoter.  Also, if any of the cases highlighted in the paper have been reported before, that should be acknowledged (such as for NAIP2, reported by reference 20 in the authors' reference list).

**Response**: We thank the reviewer for these constructive suggestions. We address each point below.

6.1) We have now provided genomic coordinates of all highlighted accessible TEs-derived TSS in figures and additional file 2, and provided genome browser session links (Fig. 2d: <https://bit.ly/3hZryCj>; Fig. 3d: <https://bit.ly/2EVYLjX>). For example, the MER90 element from which RnaseI was transcribed is located at chr1:153749308-153749588.

6.2) Based on the reviewer’s suggestion, we designed the following strategy to estimate the actual percentage contribution of the TE promoters to overall gene expression. First, we constructed complete transcripts by reference-based assembling RNA-seq data. We used stringent cutoff to remove low-confidence transcripts. Then, we intersected the TSS and the first exon of assembled transcripts to TEs locations, and further validated the TE-derived TSS by using chromatin accessibility. Of the 453 genes with TE-derived TSS, 316 genes had detectable expression in at least one tissue (>0.1 RPKM). 193 (61%) of these genes used TE-derived promoter exclusively to initiate transcription. 107 (34%) of them utilized both TE-derived TSS and non-TE TSS. Only 16 (5%) used non-TE TSS in these five tissues more exclusively. We also assessed the contribution of TE-derived TSS in producing transcripts, and found that most genes had a higher fraction of their total transcripts originated from TE-derived TSS across all five tissues and two developmental stages. To further quantify the pattern of differential usage of TE-derived promoter, we compared the expression level of transcription initiated from TE-derived promoter with the non-TE derived promoter for the 107 genes which utilized both types of promoters. A little more than half (55/107) of the genes used non-TE-derived promoters more often, and when they did so, the pattern was uniform across all five tissues. In contrast, the remaining half (52/107) used TE-derived promoters more often, and when they did so, the pattern was tissue-specific.

In addition, we analyzed the CAGE data of five matching tissues generated by FANTOM5. 114 of 453 candidate genes were detected by CAGE-seq in five mouse tissues during embryo development, and 75 of them had CAGE peaks at TE-derived promoters. By assessing the expression level of TE-derived promoter and non-TE-derived promoter for each gene using CAGE data, we found that about half of the genes only used the TE-derived promoter to initiate gene expression at both E14.5day and P0day. Importantly, we found very high concordance between CAGE-seq signal and assembled RNA-seq expression in TE-derived promoter usage.

6.3) We now present several new examples, including *Tfpi2* and *Timd2*, in more detail in the revised manuscript.

These new results are now presented in Fig. 6d,e, and Additional file 5: FigureS7.

6.4) Finally, we specifically checked the two genes (*Rnase1* and *Naip2*) reviewer mentioned. *Rnase1* showed tissue-specific usage of MER90 across the five tissues. MER90 element drove about 25% expression in the liver and about 40-60% expression in the other four tissues (fig-6c, amplified below). In contrast, *Naip2* used the canonical promoter more often, and the ORR1E element drove about 0~16% of expression across all five tissues.

**Fig. 6 Contribution of 5’ end TE-derived TSS in tissue-specific gene expression. b).** Contribution of 5’ end TE-derived TSS in gene expression at two developmental stages in five mouse tissues. Y-axis: the expression percentage of 5’ end TE-derived transcripts contributed to the total gene's expression at both E14.5 and P0 stage in each tissue.

7. In the last results section (top of page 15), the authors comment that two SINE elements around the TSS of Timd2 have binding motifs for repressive TFs, which cause them to hypothesize that TEs can also carry repressive TF binding sites to silence gene expression.  This concept is not novel as other examples of TEs being transcriptional repressors have been published.  For example, see the work of Didier Trono.

**Response**: We appreciate the reviewer’s suggestion, and updated our discussion section as:

“Previous studies indicated KRAB-ZFPs could repress the activity of LTRs through histone modification and DNA methylation, and further repressed the expression of nearby genes in both ES cells and adult tissues [90, 91]. Thus, it is tempting to hypothesize that the integration of the two SINE elements around the ancestral TSS of *Timd2* resulted in the silencing of that TSS, which indirectly facilitated the RLTR14-int-derived TSS to take over the expression of *Timd2*.”

8. Some of the figure legends are too brief and need more explanatory detail.

**Response**: We apologize, and have carefully revised our figure legends by adding more explanatory details.

**Reviewer #2:** In their manuscript "Transposable elements shape tissue-specific gene expression during mouse development", the authors use mouse ENCODE ATAC-seq and RNA-seq data to investigate TE accessibility, and their impact on gene expression across 4 different tissues.  They found that TEs were associated with over 20% of accessible chromatin regions during development, with both tissue-specific and developmental stage-specific variability.  They suggest that many of these TEs were co-opted as functional gene promoters, rewiring developmental gene expression in a tissue-specific manner, based on the transcription factor binding sites found within the TE sequences.

The scientific question they try to address is interesting and compelling.  However  the way in which the analyses were conducted is not rigorous enough to support all of the findings.  Not a single p-value is shown in the entire manuscript, since apparently they did not conduct statistical tests to support most of their findings. In particular, ~40% of the mouse genome is made of TE, so I would expect that about 1 out of 2 accessible regions would overlap a TE by chance.

For example, in Line 115 the authors write: "….In each tissue, TEs were associated with 17% (kidney) to 28% (liver) of accessible regions, and about half of these accessible TEs directly overlapped with the active histone modification H3K27ac…".  Are those percentages more than expected by chance?  How many accessible (or acetylated) regions are found overall in the kidney and in the liver? Would the difference in numbers of accessible regions across tissues affect their conclusions?

There are dozens of similar statements in the manuscript, not fully supported by statistics.

In the last few years, there were many papers investigating TE recruitment in gene regulatory networks and most of them were based on enrichment analyses rather than overlap analyses. In other words, given a set of accessible regions, how many of them are statistically enriched with TEs?  As a matter of fact, there is a computational tool designed for this scope (PMID: 23637635), which was used in most of the above mentioned papers.  This tool performs permutation tests (or binomial if preferred) to look for TE enrichment in a given set of genomics region.

To be clear, I completely understand that the main question is "how many TEs are accessible in each tissue at each time point?". And you do not need a statistical test to answer to such question.  But without statistics the comparisons between tissues and between developmental stages are not supported.

**Response**: We appreciate the reviewer for the comments and apologize for the lack of the statistical test throughout our manuscript. We completely agree that they are essential for the rigor of the study. Here, we address the reviewer’s suggestion in two parts.

Part 1: We carefully revised our manuscript and applied statistical tests to all comparisons. Specially, we updated Fig. 1e, Fig. 3b,e, Fig. 4e, Fig. 5c,e as following:

We updated the New Fig. 1e to indicate the FDR of each enriched GO terms, and also visualized the enrichment statistical significance (p-value) of all binding motifs in New Fig. 3e.

Fig. 1e	Fig. 3e

**Fig. 1 Accessible transposable elements (TEs) in five mouse tissues. e).** Gene ontology (biological process) enrichment analysis of tissue-specific accessible TEs in five mouse tissues. GO terms related to tissue-specific function were selected to visualize.

**Fig. 3** **Dynamic accessibility changes of TEs during mouse embryonic development. e).** Tissue- and developmental stage-specific enrichment of predicted transcription factors binding motifs in dynamically changed accessible TEs at E14.5 (**left**) and P0 (**right**) in five mouse tissues. The color of the dot indicated the enrichment p-value of motif; the size represented the percentage of DARs that contained the TF motif.

We perform the Wilcoxon signed-rank test to measure the significant expression difference of genes associated with the accessible TEs between E14.5 and P0 development stages in New Fig. 3b.

**Fig. 3 Dynamic accessibility changes of TEs during mouse embryonic development.** **b).** Expression pattern of genes around accessible TEs at two development stages in five mouse tissues (Wilcoxon signed-rank test).

We used the ANOVA test to measure the significant change of gene expression, including *Chit1, Havcr1, Timd2, and Rxra,* among the five tissues.

Fig. 4e

Fig. 5c	Fig. 5e

In addition, we also updated our manuscript to include statistics of co-occupancy between accessible regions and K27ac enriched regions (Additional File 4). Close to half of accessible TEs overlapped H3K27ac enriched regions across five tissues, which was similar to how non-TE accessible regions overlap with H3K27ac (table below).

Part 2: We appreciate the reviewer’s understanding that the question we tried to address is “how many TEs are accessible in each tissue at each time point?". Thus, we did not specifically address whether TEs are enriched or depleted for accessible regions. In fact, our expectation was that TEs should be depleted of accessible chromatin, as the reviewer pointed out from the observed numbers. The worthwhile discussion here is, what is the null hypothesis? If we use the random genomic background as an expectation, as most analyses do, we would clearly see the depletion of accessible chromatin from TEs, and this is consistent with the general notion that most TEs are epigenetically silenced. However, if our null hypothesis is “TEs are epigenetically silenced”, then the identification of accessible TEs is indeed a surprise, or biologically significant (if not statistically significant). This point has been discussed many times in the literature, including in a few of our own publications [2-4]. And we believe most investigators are in agreement with the notion that TEs are depleted for active elements, but they still contain a substantial amount of active elements due to their large genome occupancy. The sheer number is large enough to require us to not ignore them, but to investigate their functional relevance.

That said, we completely agree with the reviewer that we should present relevant statistics and make this message clearer. Thus, we followed the reviewer's suggestion and estimated the relative enrichment (or depletion) of accessible chromatin in TEs. Briefly, we followed the method of Kapusta, et al. 2013 [5]. We applied GenometriCorr to calculate the correlation between TEs and accessible regions identified in our study. GenometriCorr is an R package that used to calculate the spatial correlation of genome-wide interval datasets with the null hypothesis: interval sets are spatially independent. The correlated interval sets would follow a detectable pattern of the location, such as being consistently nearby or far away from each other in genomic coordinates, or preferentially overlapping.

By using GenometriCorr, we applied four statistical tests, including Kolmogorov-Smirnov test, permutation tests, projection test, and Jaccard measure, to measure the spatial correlation between accessible regions and TEs. All those statistic tests indicated a significant lack of overlap between all accessible regions and all TEs in all five tissues, although a different number of accessible regions were identified in those 5 mouse tissues (results in Additional file 3). Specifically, the very low value of “relative.distances.ks.p.value” and negative value of the “relative.distances.ecdf.area.correlation” showed accessible regions and TEs were, in general, separated from each other. The “projection.test.p.value” was zero, and “projection.test.lower.tail” was TRUE, both indicating a significant lack of overlap between accessible regions and TEs than expected if they were independent. All three permutation tests give <0.01 which means that observed spatial relationships were significantly different from what was observed in the permutation distribution. The value of the “scaled.absolute.min.distance.sum.lower.tail” was TRUE, showing the distances between accessible regions and TEs are consistent and small, and the “jaccard.measure.lower.tail” was TRUE, indicating an unexpected lack of overlap.

Thus, all these results suggested the accessible regions in five mouse tissues were not enriched in TEs, and quite contrary to this, accessible regions were depleted in TEs in the mouse genome.

Additional file 3

The second major issue I found while reading the manuscript is that the methods are not described in a rigorous way.  The authors mention the softwares used for each step of the analysis, but they never include any parameter For example in line 393 the sentence: "During the processing of RNA-seq data, we used Cutadapt, FastQC, and STAR to do the trimming, QC report, and genome mapping processes [74-76]."  is lacking details.

Even worse, the sentence: "We also got the expression of some human genes in the 5 tissues from NCBI gene database" is not up to Genome Biology standard (or to any scientific publishing standard to be honest)

**Response**: We sincerely apologize for not providing sufficient methodology details. We have carefully revised our manuscript to include all the necessary details.

“RNA-seq data of five tissues with two development stages were processed by Cutadapt (v2.7; --quality-cutoff=15,10 --minimum-length=36), FastQC (v0.11.4), and STAR (v2.5.2b; --quantMode TranscriptomeSAM --outWigType bedGraph --outWigNorm RPM) to do the trimming, QC report and mouse genome mapping (mm10) [97-99]. Then, the mouse gene expression was calculated by featureCounts (-p -T 4 -Q 10) [100] based on GENCODE vM20 gene annotation. The expression (TPM) of CHIT1 and HAVCR1 in those five human tissues were obtained from "GTEx_Analysis_2017-06-05_v8_RNASeQCv1.1.9_gene_tpm.gct" file, which was processed and normalized by GTEx Portal (https://www.gtexportal.org/home/)[101].”

Minor comments:

- Line 138: Did the authors perform a motif analysis for TEs constitutively accessible to understand what is determining the widespread accessibility of these TEs across tissues?

**Response**: We thank the reviewer for a constructive suggestion, and have performed TFBS analysis following the suggestion. We identified enriched CTCF, Usf1, and Klf4 binding motifs in constitutively accessible TEs, and have provided this new result as Additional file 4.

- Line 163: Does developmental stage changes in TE accessibility correspond to change in expression of the associated gene?  The authors should be able to address this using the RNA-seq data they have downloaded.

**Response**: Following the reviewer’s suggestion, we assigned E14.5day-specific and P0day-specific accessible TEs to their nearest genes in 5 tissues, and further compared the expression differences of these putative target genes between the two development stages. Consistent with the reviewer’s expectation, for most of the comparisons we observed developmental stage-specific gene expression patterns that are consistent with the developmental stage-specific chromatin accessibility pattern. This new result is now included in new Fig. 3b. We updated our result as:

“Genes associated with developmental stage-specific accessible TEs generally also displayed developmental stage-specific expression pattern (Fig. 3b). Since ~95% of developmental stage-specific accessible TEs were distal to gene's promoter, our results supported previous findings that TEs can play roles as a distal enhancer in gene regulatory networks (Additional file 8: Table S2) [9, 34, 48, 56]”

**Fig. 3 Dynamic accessibility changes of TEs during mouse embryonic development.** **b).** Expression pattern of genes around accessible TEs at two development stages in five mouse tissues (Wilcoxon signed-rank test).

- Did the authors perform any comparison of their liver findings with the findings of a recent study the found significant TE recruitment in the primate liver gene regulation (PMID: 28855262)? Did they observe any conserved TE with a function in the liver gene regulation of both mouse and primates?

**Response**: We thank the reviewer for raising an interesting question. Although most TEs in the human and mouse genomes are specific to each lineage, the two species do share some older TEs. Following the reviewer’s suggestion, we compared subfamily-level enrichment results of accessible TEs (Fig. 1g) in our study to the previous study [6](PMID: 28855262; Figure 6) that tested the regulatory ability of TE families in primate liver. Interestingly, the TE subfamily LTR41B was found to be enriched in both mouse liver and human liver cell line HepG2. We have added this interesting result to our manuscript:

“LTR41b, an LTR subfamily exhibiting strong regulatory ability in human liver cancer cell line HepG2 [55], was highly accessible in four mouse tissues but not the intestine.”

References

1.	Thompson PJ, Macfarlan TS, Lorincz MC: **Long terminal repeats: from parasitic elements to building blocks of the transcriptional regulatory repertoire.** *Molecular cell* 2016, **62:**766-776.

2.	Pehrsson EC, Choudhary MN, Sundaram V, Wang T: **The epigenomic landscape of transposable elements across normal human development and anatomy.** *Nature communications* 2019, **10:**1-16.

3.	Sundaram V, Cheng Y, Ma Z, Li D, Xing X, Edge P, Snyder MP, Wang T: **Widespread contribution of transposable elements to the innovation of gene regulatory networks.** *Genome research* 2014, **24:**1963-1976.

4.	Sundaram V, Wang T: **Transposable Element Mediated Innovation in Gene Regulatory Landscapes of Cells: Re‐Visiting the “Gene‐Battery” Model.** *BioEssays* 2018, **40:**1700155.

5.	Kapusta A, Kronenberg Z, Lynch VJ, Zhuo X, Ramsay L, Bourque G, Yandell M, Feschotte C: **Transposable elements are major contributors to the origin, diversification, and regulation of vertebrate long noncoding RNAs.** *PLoS Genet* 2013, **9:**e1003470.

6.	Trizzino M, Park Y, Holsbach-Beltrame M, Aracena K, Mika K, Caliskan M, Perry GH, Lynch VJ, Brown CD: **Transposable elements are the primary source of novelty in primate gene regulation.** *Genome research* 2017, **27:**1623-1633.

**Second round of review**

**Reviewer 1**

The authors have adequately addressed my concerns and have strengthened the manuscript.

**Reviewer 2**

The author addressed all of my concerns.
